# Role of Vibrational-Assisted Scattering and Surface-Enhanced Raman Scattering in Colloidal Plexcitonic Materials

**DOI:** 10.1021/acsnano.4c17571

**Published:** 2025-04-16

**Authors:** Nicola Peruffo, Minpeng Liang, Rahul Bhuyan, Vajradhar Acharya, Johanna L. Höög, Karl Börjesson

**Affiliations:** † Department of Chemistry and Molecular Biology, University of Gothenburg, 413 90 Göteborg, Sweden; ‡ Department of Applied Physics and Science Education, Eindhoven Hendrik Casimir Institute and Institute for Complex Molecular Systems, Eindhoven University of Technology, 5612AE Eindhoven, The Netherlands

**Keywords:** plasmon, plexciton, polariton, SERS, vibration, cavity, emission

## Abstract

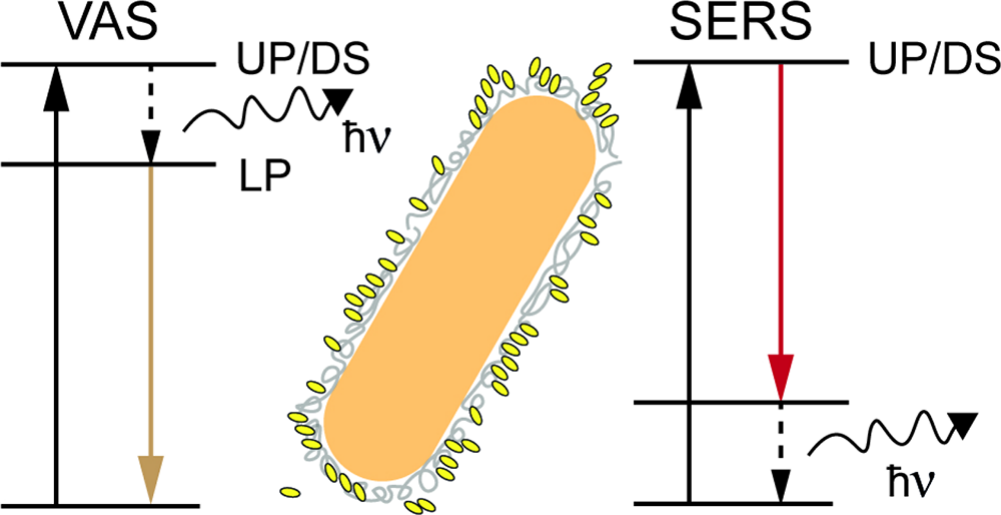

Strong coupling between excitons and an electromagnetic mode leads to the formation of polaritonic materials. These half-light half-matter states obey Bose–Einstein statistics and have therefore promised a route toward room temperature condensates and low-threshold polariton lasers. However, our understanding of how to enhance the rate of relaxation toward the lowest energy excited state must be greatly enhanced for electrically driven organic condensates and polariton lasers to be realized. Here, the mechanism of excited-state relaxation in colloidal plexcitonic materials (CPMs) is explored. CPMs are a subgroup of polaritonic materials formed when an exciton interacts strongly with a plasmonic resonance of a nanoparticle. Based on our current understanding of relaxation in polaritonic systems, which is based on experiments done using Fabry–Pérot cavities, CPMs are expected to have high relaxation rates through the vibrationally assisted scattering (VAS) mechanism. However, so far, it has been unclear whether we can transfer the knowledge gained from Fabry–Pérot cavities to plasmonic cavities. Our results indicate that not only VAS but also surface-enhanced Raman scattering (SERS) is active in CPMs and that the predominant mechanism depends on to which state excitation occurs. Therefore, caution must be exercised when interpreting the emission from plexcitonic materials and when using theories obtained from polaritonic materials prepared with Fabry–Pérot cavities on plexcitonic materials. Additionally, we found that plexcitonic materials can provide an electromagnetic enhancement of both the excitation and emission part in SERS, increasing its enhancement factor and allowing tuning of the sensitivity to specific vibrations.

## Introduction

Enhanced electromagnetic fields can couple strongly to excitons, forming polaritonic materials and thereby changing the molecular properties without synthetic modifications.^1^ These polaritonic materials can effectively alter reaction rates,^2−4^ and show promising potential to be used for optoelectronics and quantum technologies due to their quantum coherences,^5^ long energy migration paths^6,7^ and condensation behavior.^8,9^ Polariton condensation offers the attractive promise of low threshold lasing. However, a limiting factor is in the rate at which the excited-state energy is relaxed in such systems. Therefore, a better understanding of how to enhance energy relaxation in polaritonic materials is a prerequisite for the construction of functional polaritonic materials.

**Figure 1:**
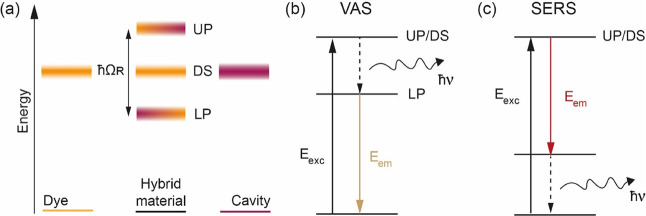
(a) Energetic landscape of strong coupling. A molecular transition strongly couples with a light mode enhanced by a cavity (a Fabry–Pérot or plasmonic cavity). A polaritonic/plexcitonic material is generated, comprising of an upper polariton/plexciton (UP), a lower polariton/plexciton (LP), and a manifold of dark states (DS). (b) Vibrational-assisted scattering mechanism (VAS). The relaxation from the UP or the DS is mediated through the activation of a vibration with energy *ℏ*ν. (c) Surface-enhanced Raman scattering mechanism (SERS). The light scatters from the UP or the DS, activating a vibration of the ground state with energy *ℏ*ν.

In polaritonic materials, *N* excitons and an electromagnetic mode couple strongly to produce two hybrid light–matter states, the upper and lower polaritons (UP and LP), which energy splitting is called the Rabi splitting (*ℏ*Ω_R_). Furthermore, the remaining *N*-1 states in the energetic landscape have (almost) no oscillator strength and are therefore often denoted as dark states (DS; or the exciton reservoir; Figure 1a).^10^ The electromagnetic mode is provided by scaffolds called cavities. Most of our knowledge about polaritons, including their mechanism of relaxing energy, comes from polaritonic materials prepared with Fabry–Pérot cavities. Here, an arrangement of mirrors forms a standing waves resonator for the electromagnetic field (E-field), which is consequently enhanced.^11^ Radiative pumping and vibrational-assisted scattering (VAS; Figure 1b) are the two main relaxation mechanisms toward the LP.^12,13^ These two phenomena increase the relaxation toward the LP and thus enhance its emission intensity. Radiative pumping involves a virtual photon emitted by a DS that is absorbed by the LP, and it predominantly occurs for molecules with large Stokes’ shifts. On the contrary, VAS is the predominant relaxation mechanism for molecules with small Stokes shifts,^14^ and it mediates relaxation through the scattering of molecular vibrations.^15^ VAS has so far only been seen using intense light sources such as lasers and has been observed to be maximized when the molecular vibration matches the energy gap between the DS^16^ (or the UP)^17^ and the LP. Additionally, the rate of VAS has been shown to be inversely proportional to the number of excitons, *N*, coupled to the cavity mode.^13,18^ Thus, the VAS mechanism is expected to become more dominant and possibly faster than other relaxation rates in cavities having smaller *N*, which would increase the rate of energy relaxation toward the LP, of importance for reducing the lasing threshold in polaritonic lasers.

While Fabry-Pérot cavities have values of *N* on the order of 10^4–6^,^19^ plasmonic nanoparticles can accommodate only 10–100 *N*.^20−22^ Thus, the rate of VAS is expected to become more predominant when using nanoparticles compared to Fabry-Pérot cavities. However, much less is known about plasmonic cavities. Nanoparticles can amplify the E-field through their plasmonic resonances,^23^ and the subgroup of polaritonic materials prepared with nanoparticles are called plexcitonic materials.^24,25^ In analogy to the polaritonic energy landscape, they possess an upper and a lower plexciton (UP and LP), separated by the Rabi splitting *ℏ*Ω_R_, as well as a manyfold of dark states (DS).^26−29^ Although both are hybrid light–matter materials, polaritonic and plexcitonic materials differ in how and where their cavities amplify the E-field. Standing waves in Fabry-Pérot cavities are delocalized along the thickness of the entire cavity (0.1–1 μm). In contrast, plasmonic resonances of single nanoparticles enhance the E-field on the order of 10 nm from their surface.^21^ Thus, only a few excitons can be strongly coupled in this small volume.^20^ Although this could be beneficial for increasing VAS, it also significantly increases the E-field enhancement and thus also favors Raman scattering. This phenomenon is called surface-enhanced Raman scattering (SERS; Figure 1c), and it is widely used to detect chemical and biological analytes.^30^ Intense light sources are generally used to measure SERS,^30^ which has also been observed in plexcitonic materials when exciting UP,^31,32^ LP,^33−36^ or the DS.^33,37^

The differences between cavities make it unclear if it is possible to translate our understanding of the relaxation processes of the excited states from Fabry–Pérot cavities to plasmonic cavities. Using low-*N* plasmonic cavities to increase relaxation rates and thus increase the propensity of polariton lasing from the LP seems to be a viable idea. However, it is necessary to first understand how VAS and SERS contribute to the apparent plexcitonic emission. To achieve this goal, we have prepared colloidal plexcitonic materials (CPMs),^24^ in which colloidal nanoparticles were synthesized by wet chemistry and dispersed in solution. We analyzed the emission of the CPMs and compared their nature to that of strongly coupled Fabry–Pérot cavities, widely documented in the literature.^1,11−17,38−41^ SERS and VAS models were used to interpret the data. We found that, while neither processes were active in Fabry–Pérot cavities at low-intensity excitation conditions, both processes were active in CPMs. However, the effect of the processes is maximized under different conditions that depend on the Rabi splitting, the excited state (the UP or the DS), the value of *N*, and the set of vibrations of the coupled molecules. Thus, it is important to pay attention to the conditions used to not enhance SERS by mistake. We present here a framework to distinguish spectral signatures of SERS from VAS, of crucial importance when optimizing energy relaxation in plexcitonic systems. Understanding the effect of SERS and VAS using nanoparticles as plasmonic cavities is important to also understand the behavior of other plasmonic cavities such as plasmonic arrays, where condensation has been observed,^8,42^ and the number of coupled excitons is small as well, in the order of 10^3^.^43^

## Results and Discussion

### Preparation of CPMs

**Figure 2:**
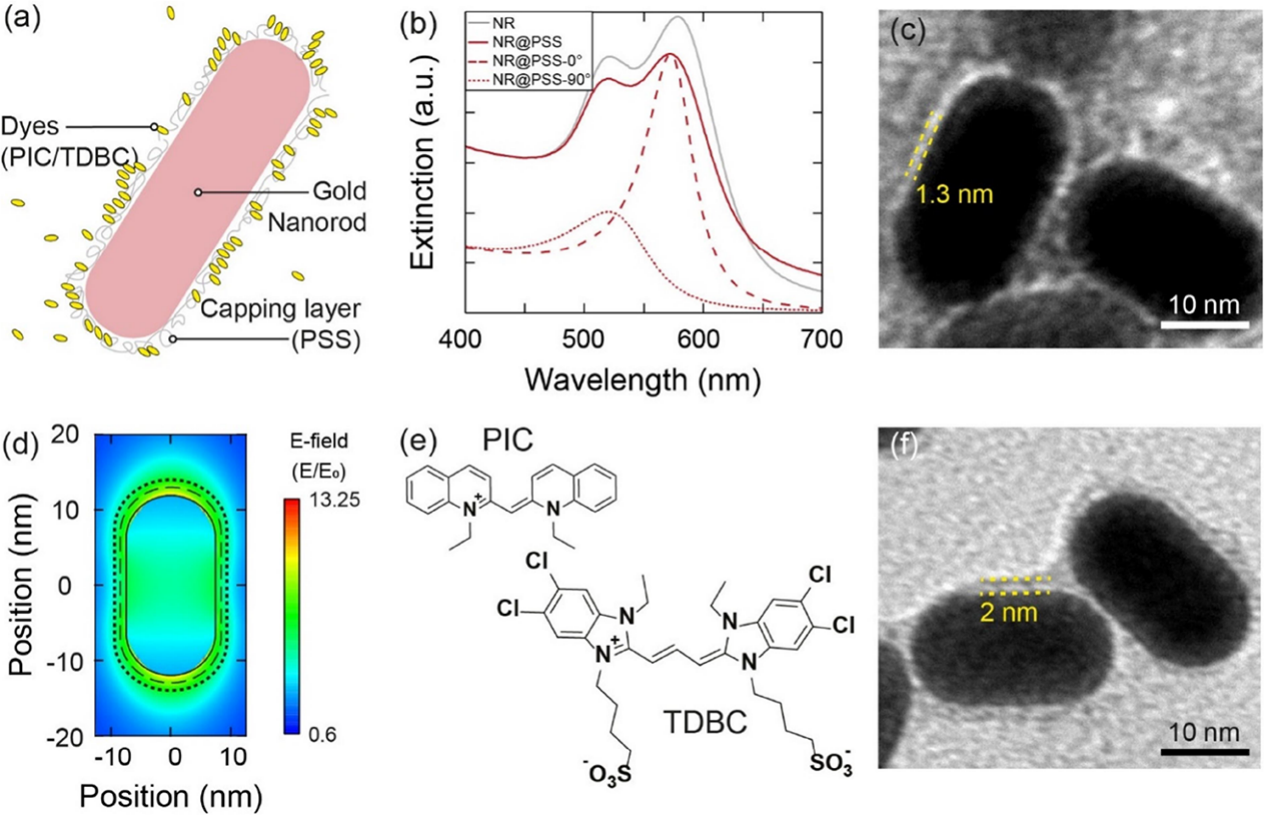
(a) Design of the analyzed CPM, composed of a gold nanorod (NR), a PSS capping layer, and dye molecules (PIC or TDBC J-aggregates). (b) Normalized extinction spectra of the NR covered with CTAB (gray solid line), and PSS (red solid line) using unpolarized light. Simulated extinction spectra of NR@PSS when exciting with light polarization along the NR long axes (dashed line), and along the short axes (dotted line). (c) TEM micrograph of the NR@PSS. (d) E-field of the central vertical section of the NR@PSS. The area between the solid and the dashed lines defines the PSS layer (1 nm thick), while the area between the dotted line and the inner solid line defines a 2 nm-thick capping layer. (e) Molecular structure of TDBC and PIC. (f) TEM micrograph of NR@PSS after the addition of PIC. The extinction of the CPM is reported in Figure S9a.

CPMs were selected to investigate the role of VAS and SERS in plexcitonic materials because their structure is suitable for accommodating small values of *N*. In addition, CPMs can be prepared with J-aggregates of cyanine dyes, which have been extensively used to study VAS in Fabry–Pérot cavities.^14,16,17,41^ A J-aggregate is an ordered assembly of molecules, where the excited states of several molecules couples together to form one exciton. The number of excitons is therefore smaller than the number of molecules. Furthermore, molecules not assembled into the J-aggregate are here denoted as free molecules. The structure of CPMs can be divided into three parts: (i) the nanoparticle, which acts as a plasmonic cavity; (ii) the molecules that couple to the nanoparticle; and (iii) the capping layer, a molecular layer that encloses the nanoparticle, preserving its stability in solution and providing the binding to the molecules (see Figure 2a).^24^ This assembled material is defined as a CPM if its *ℏ*Ω_R_ overpasses the half-width at half-maximum of the plasmonic/molecular extinction peak, *ℏ*κ and *ℏ*γ, which are the equivalent in energy to the rates of energy dissipation for the plasmonic resonance and the molecular transition, respectively.^24,44^

#### Preparation of Gold Nanorods

Gold nanorods (NRs) were used as plasmonic cavities due to their small *ℏ*κ compared to other plasmonic nanoparticles^45,46^ and high surface-to-volume ratio,^24^ making them suitable plasmonic nanoparticles for plexcitonic materials. The NRs were synthesized using a seeded growth protocol,^47^ and subsequently oxidized in the presence of HAuCl_4_ and cetyltrimethylammonium bromide (CTAB),^48^ which also acts as a capping layer. The detailed procedure is described in the Experimental and Methods section. The NRs exhibit a longitudinal plasmon at 578 nm and a transversal plasmon at 515 nm (Figure 2b). The NRs were covered with polystyrenesulfonate (PSS) for its ability to bind several dyes, among which those used in this work, and produce plexcitonic materials.^49−52^ The zeta potential of the NRs before adding PSS was measured to be 14 meV. The positive value is because CTAB is cationic. The zeta potential of the NRs dropped to −24 meV after coating with PSS (NR@PSS), because of its anionic nature. The successful addition of PSS was also confirmed by the blueshift of the longitudinal plasmon extinction, from 578 to 572 nm (Figure 2b), in line with previous reports.^49^ Negative stain transmission electron microscopy (TEM) revealed that NR@PSS had a diameter of 14.5 ± 1.8 nm, a height of 24.2 ± 5.3 nm, and a capping layer thickness of 1.3 ± 0.2 nm (Figures 2c, S3, S5a, and S5b). The distribution of the NRs aspect ratios was 1.71 ± 0.31 (Figure S3e). From the aspect ratio, the distribution of the longitudinal plasmon wavelengths was calculated, according to the relationship:^53^ λ_Longitudinal plasmon_ = (53.71 × Aspect Ratio – 42.29) × 1.33^2^ + 495.14. The predicted plasmon resonance is centered at (583 ± 30) nm (Figure S3f), which is in agreement with the longitudinal plasmon measured in the extinction spectrum (572 nm). Furthermore, thicker PSS layers on NRs can be prepared by adding a solution with an increased concentration of PSS (see Supporting Information, Section S2).

The optical properties of the NRs were simulated using the finite-difference time-domain method (FDTD method, Lumerical FDTD software, see also Experimental and Methods section). The simulated extinction spectra are consistent with the experimental extinction of the NR@PSS for both the longitudinal and transverse plasmons (Figure 2b). Furthermore, the enhancement of the NR@PSS E-field is halved at 2 (3.5) nm in the lateral (apical) direction to the surface and is thus partially confined in the PSS layer (Figure 2d, dashed lines).

#### Coupling NR@PSS with PIC and TDBC J-Aggregates

The VAS mechanism has been studied in Fabry–Pérot cavities using J-aggregates of 5,6-dichloro-2-[[5,6-dichloro-1-ethyl-3-(4-sulfobutyl)-benzimidazol-2-ylidene]-propenyl]-1-ethyl-3-(4-sulfobutyl)-benzimidazolium hydroxide, inner salt, sodium salt (TDBC).^14,16,17,41^ Moreover, 1,1′-diethyl-2,2′-cyanine iodide (PIC) and TDBC (Figure 2e) have both been abundantly employed for the preparation of CPMs.^24,54−56^ PIC and TDBC have a low ℏγ, and they can bind to nanoparticles covered with PSS to produce plexcitonic materials.^50,57^ The addition of PIC to a NR@PSS solution swelled the thickness of the capping layer from 1.3 ± 0.2 to 1.9 ± 0.8 nm (Figures 2f, S5a,b, and S6). Additionally, the zeta potential increased to −11 and −7 meV for NR@PSS bound with PIC and TDBC, respectively. These two pieces of information indicate that the PSS layer binds TDBC and PIC close to the NR surface, where the E-field is particularly enhanced (Figure 2d, dotted lines) favoring coupling between the plasmon and the transition dipole moment of the dyes.

**Figure 3:**
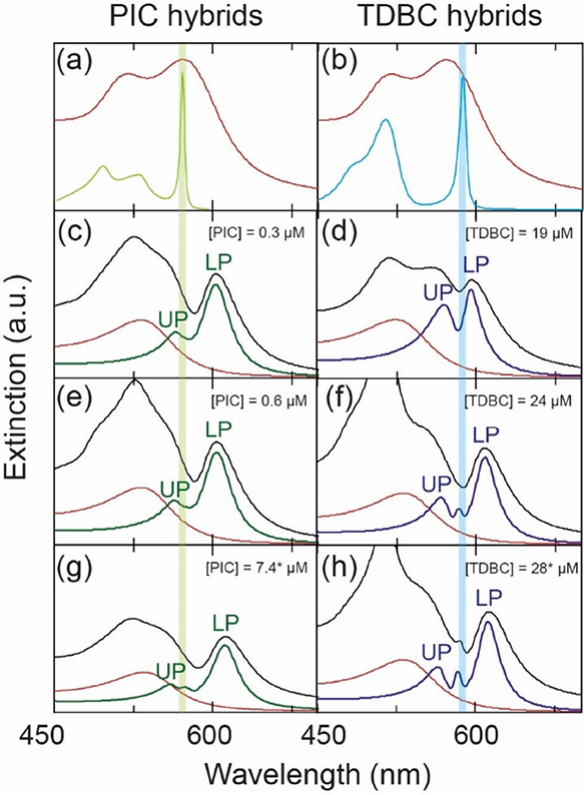
(a) Experimental extinction spectra of the NR@PSS (red line) and experimental absorption of the J-aggregate of PIC (green line). (b) Experimental extinction spectra of the NR@PSS (red line) and experimental absorption of the J-aggregate of TDBC (cyan line). In all the panels, a colored vertical line indicates the excitonic wavelength of the J-aggregates. (c) Experimental extinction of CPMs prepared with [PIC] = 0.3 μM (black lines), and simulated extinction with polarization along the short (dark red) and long axes (dark green). The same spectra are reported for (e) and (g), increasing [PIC] to 0.6 μM and 7.4 μM, respectively. (d) Experimental extinction of CPMs prepared with [TDBC] = 0.3 μM (black lines), and simulated extinction with polarization along the short (dark red) and long axes (dark blue). The same spectra are reported for (f) and (h), increasing [TDBC] to 24 and 28 μM, respectively. For [PIC] = 7.4 and [TDBC] = 28 μM, the extinction of the CPMs is reported after a washing step and the concentrations are labeled with an asterisk.

The NR@PSS was mixed with solutions of PIC or TDBC and the extinction spectra of the CPMs obtained were acquired (Figure 3c–h). TDBC and PIC J-aggregates are expected to couple with the longitudinal plasmon of NR@PSS because they absorb in the same wavelength region. Indeed, the UP and LP appeared by increasing the PIC concentration from 0 to 7.4 μM in the NR@PSS dispersion and the *ℏ*Ω_R_ increased up to 190 meV (1512 cm^–1^). The same happened when varying the concentration of TDBC, from 0 to 28 μM, with a final *ℏ*Ω_R_ of 200 meV (1608 cm^–1^). However, the UP was partially obscured due to an increased signal in the 510–530 nm region. This signal comes from free molecules, which are molecules not in J-aggregate form, that absorb in a region far from the longitudinal plasmon, and thus are unable to strongly couple with it. For this reason, the [PIC] = 7.4 and [TDBC] = 28 μM samples were washed through centrifugation prior to measuring the extinction spectrum. In total 20 CPMs were made by using different NR batches, PSS loadings, and dye concentrations, of which 6 are presented in the main manuscript, and the data of the remaining are shown in the Supporting Information (Table S1 and Figures S7–S11). The UP and the LP of these CPMs are distributed around 550–565 nm (2.254 × 10^4^–2.194 × 10^4^ cm^–1^) and 590–615 nm (2.101 × 10^4^–2.016 × 10^4^ cm^–1^), respectively. The DS region can roughly be attributed to the dip in extinction between the UP and the LP (579 and 587 nm; 1.727 × 10^4^ and 1.704 × 10^4^ cm^–1^).

FDTD simulations confirmed the presence of the UP and the LP in both PIC and TDBC plexcitonic materials (Figures 3, S12, and S13). The amount of dye added to the NR@PSS solutions was higher for TDBC than for PIC to achieve a spectrally resolved *ℏ*Ω_R_, which is in line with previous reports.^57^ This difference is likely due to different binding constants to the NR@PSS. The extinction spectra of the uncoupled NR@PSS and the absorption spectra of TDBC and PIC J-aggregates were used to calculate *ℏ*κ (90 meV) and *ℏ*γ (30 meV) for both dyes. Thus, the *ℏ*Ω_R_ for all CPMs exceeded the dissipative rates, confirming their plexcitonic nature.

### Emission Spectra of CPMs

The contribution of VAS and SERS can be evaluated by studying the emission of strongly coupled materials. Radiative pumping can be excluded because it is present mostly in strongly coupled dyes with large Stokes’ shifts, while J-aggregates have generally very small Stokes’ shifts.^14^ VAS is expected to increase the population of the LP and thus its emission, whereas SERS is measured by collecting the scattered photons and it can contribute to an apparent increase in the emission. To appreciate both mechanisms, it is necessary to plot the emission as a function of the difference between the excitation and emission energies (*E*_exc_ – *E*_em_). This is because both VAS and SERS are mechanisms that activate a vibration whose energy ℏν corresponds to *E*_exc_ – *E*_em_ (Figure 1b,c), and consequently increases the (apparent) emission.

The emission spectra of the CPMs were collected exciting in the region between the transversal plasmon and the LP (505–610 nm) with a step of 5 nm. The emission was scanned with a step of 1 nm up to 690 nm. For CPMs prepared with TDBC, the excitation window was reduced to 540–610 nm because the emissive species in solution produced an emission too intense to separate contributions from VAS and/or SERS. The literature reports several possible emissive species, such as free molecules, J-aggregates, the LP,^16,18,58^ the DS,^59,60^ and, more rarely, the UP.^61^ In our case, the most important contribution was due to free molecules of PIC and TDBC (Figure S13). The component in the emission spectra due to the emissive species was considered as a background and removed (Figure S15).

We want to stress that the background emission follows Kasha’s rule as it is wavelength-independent. In the background emission, the only contribution that can be detected comes from free molecules. Consequently, any emission from the LP that follows Kasha’s rule can be ruled out, which typical for VAS reported in many recent papers.^14,62^ In that case, a laser excites the polaritonic sample at energies higher than the UP. The sample absorbs the radiation and relaxes rapidly to the DS, from which VAS occurs, populating the LPs and enhancing their emission. However, VAS can also occur directly after exciting a polaritonic state, when this very state scatters to the LP before any other relaxation happens.^63,64^ This can occur if the VAS rate is faster than the others, which might be made possible by the small *N* numbers present in CPMs. In this case, VAS does not follow Kasha’s rule and cannot be detected in the background emission. This is because it can be activated only if the difference between the excitation energy and the emitted photon energy equals the energy of a molecular vibration (*E*_exc_ – *E*_em_ = ℏν), which makes the phenomenon wavelength-dependent. Ultrafast surface-enhanced fluorescence^31^ breaks Kasha’s rule as well, but we can exclude that since it usually leads to very broad emissions, while VAS-enhanced emission is usually narrow as it occurs only when *E*_exc_ – *E*_em_ = ℏν.

**Figure 4:**
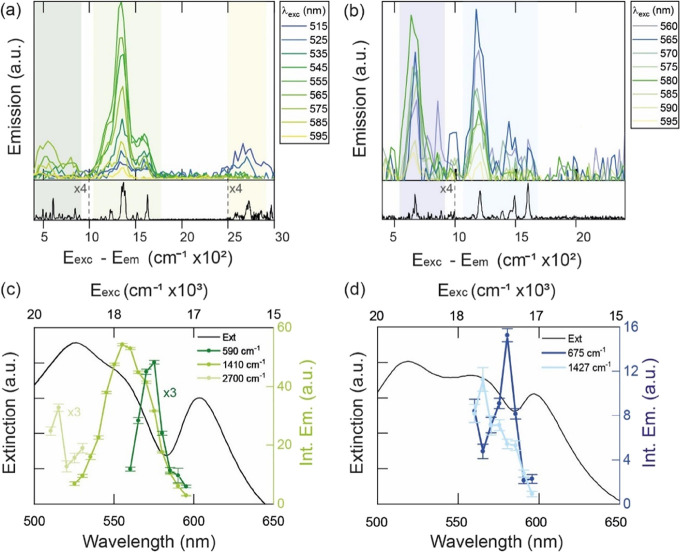
(a) Emission spectra in function of *E*_exc_ – *E*_em_ of NR@PSS coupled with PIC ([PIC] = 0.3 μM) after background removal. The excitation spectra are plotted only where the VAS/SERS signals were present, and every 10 nm for clarity. The Raman spectrum of PIC is plotted below, with the regions below 1000 cm^–1^ and above 2500 cm^–1^ are scaled by a factor of 4. Three main vibrational regions are colored. (b) Same as for (a), but for a CPM prepared with NR@PSS and TDBC ([TDBC] = 19 μM). (c) Integrated emission of the signals from the region around *E*_exc_ – *E*_em_ = 590 cm^–1^ (dark green area, scaled by a factor of 3), *E*_exc_ – *E*_em_ = 1410 cm^–1^ (green area), and *E*_exc_ – *E*_em_ = 2700 cm^–1^ (light green area, scaled by a factor of 3) in function of the excitation wavelength. The extinction spectrum of the CPM is superposed. (d) Same as for (c) but for the CPM prepared with NR@PSS and TDBC ([TDBC] = 19 μM). The integrated areas in this case are for the region around 675 cm^–1^ (dark blue) and 1427 cm^–1^ (light blue).

The resulting emission spectra of two CPMs prepared with PIC and TDBC, plotted in function of *E*_exc_ – *E*_em_, are reported in Figure 4a,b, respectively. The emission starkly resembles the Raman spectra of the uncoupled J-aggregates, reported below each panel. This supports the presence of VAS and/or SERS mechanisms being active. On a first approximation, the VAS and SERS signals will always appear at the same energy corresponding to *E*_exc_ – *E*_em_ = ℏν, regardless of the *E*_exc_ of the experiment. For the CPM prepared with PIC, the emission is enhanced when *E*_exc_ – *E*_em_ corresponds to three main PIC vibrational regions, centered at *ℏ*ν = 590, 1410, and 2700 cm^–1^. The emission of the CPM prepared with TDBC is instead enhanced in two regions, centered at *ℏ*ν = 675 and 1427 cm^–1^. Notably, the emission ratio between the regions’ changes depending on *E*_exc_, as shown in Figure 4a,b.

Successively, the emission was integrated into three/two regions corresponding to the main PIC/TDBC vibrational regions to include the contribution from all the vibrations. This process was iterated for each *E*_exc_, and for each CPM. The integrated emission for each *E*_exc_ and region is reported in Figure 4c,d, for the two CPMs prepared with PIC and TDBC, respectively (see Section S4 in Supporting Info for more details). The extinction spectra of the respective CPMs are superimposed in these figures. Figure 4c,d demonstrates that vibrations corresponding to *E*_exc_ – *E*_em_ ≈ 1400 cm^–1^ are activated when exciting the region of the UP, while vibrations at around 600–700 cm^–1^ are turned on when exciting the region of the DS. This phenomenon was observed in the analysis of 20 different CPMs using various NR batches, PSS loadings, and dye concentrations (Figures S16–S33). It is important to note that the light source used for the emission experiments was a weak Xe lamp, rather than the intense laser source commonly used in the literature for measuring SERS and VAS. As a result, no SERS signals were found for PIC nor TDBC that were weakly coupled (due to detuning) to NR@PSS (Section S5). Based on these findings, we can exclude that SERS or VAS signals originated from weakly coupled molecules. In conclusion, SERS and/or VAS are active in CPMs, even using a weak Xe lamp, and their origin is due to the plexcitonic states.

### Understanding the Relaxation Mechanisms

#### Vibrational-Assisted Scattering (VAS)

A more detailed analysis is necessary to determine whether SERS and/or VAS is responsible for the observed patterns in the emission spectra. To do so, the rate of VAS was modeled as reported by Michetti et al.^13^ According to this model, the VAS scattering rate from the DS to LP and from UP to LP, *W*_VAS_, can be calculated as follows:
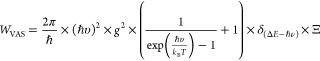
1Where ℏν is the energy of the vibration, *g* is the polariton-vibration coupling, *k*_B_ is the Boltzmann constant, *T* is the temperature, δ_(Δ*E*–ℏν)_ is a Dirac delta function that maintains the VAS condition *E*_exc_ – *E*_em_ = *ℏ*ν, and Ξ is the spatial overlap between the wave functions of the DS (or the UP) and the LP. The value of *g* was set to 0.5 for all vibrations, which is a similar value to that previously used for TDBC vibrations.^13^ δ_(Δ*E*–ℏν)_ takes into account that the scattering of a vibration due to VAS only can occur when *E*_exc_ – *E*_em_ = *ℏ*ν, or, rearranging the equation, when (*E*_exc_ – *E*_em_)/(*ℏ*ν) = 1. Since VAS can occur when either exciting the UP (*E*_exc_ = *E*_UP_) or the DS (*E*_exc_ = *E*_DS_), and the emission due to VAS occurs from the LP (*E*_em_ = *E*_LP_), it is possible to demonstrate that VAS occurs when ℏΩ_R_/(ℏν) = 1 (exciting the UP) or 2 (exciting the DS). The δ_(Δ*E*–ℏν)_ is thus always zero, except when ℏΩ_R_/(ℏν) = 1 or 2. However, this condition was relaxed due to the inhomogeneities in the NR dimensions, PSS thickness, and dye loading, which increase the inhomogeneous broadening of the plexcitonic states. Therefore, δ_(Δ*E*–ℏν)_ was set to 1 between ℏΩ_R_/(ℏν) = 1 and 2, and decreased monotonically to 0, when ℏΩ_R_/(ℏν) reaches 0 and 3 (Figure S36).

The spatial overlaps for the DS to the LP relaxation, Ξ_DS→LP_, and for the UP to LP relaxation, Ξ_UP→LP_, were calculated as^13^
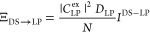
2
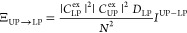
3where |*C*_UP/LP_|^2^ are the Hopfield coefficients of the UP/LP, set to 0.5 because of the zero detuning, *D*_LP_ is the number of LP states, set to 1, and *N* is the number of coupled excitons. *I*^DS-LP^ is the overlap between the DS wave function and the excitonic component of the LP wave function, while *I*^UP-LP^ is the overlap between the excitonic component of the UP and the LP wave functions. The UP, the DS, and the LP are mainly delocalized on the molecular region that covers the surface of the NR, since both the E-field of the NRs and the J-aggregates are there. Consequently, their excitonic component is localized on the surface of the NRs as well, and *I*^UP-LP^ and *I*^DP-LP^ can be set equal to one. Since |*C*_UP/LP_|,^2^*I*^UP-LP^, *I*^DS-LP^, and *D*_LP_ are constants, *N* is the only parameter that can significantly affect the spatial overlaps Ξ_UP→LP_ and Ξ_DS→LP_. The excess of free molecules makes it difficult to retrieve the value of *N* from experimental measurements. An alternative approach based on FDTD simulations was therefore used. In FDTD simulations, the exciton absorption is simulated as a Lorentzian function, where the oscillator strength parameter represents the absorption amplitude (see Experimental and Methods for more details). The oscillator strength per exciton was taken from literature values. The literature total oscillation strength was divided by the total number of excitons, giving an average value of the oscillator strength per exciton of 0.003 for PIC J-aggregates^22^ and of 0.001 for TDBC.^20,21^ The *N* value was then calculated by dividing the total oscillator strength of the CPMs simulated in this work by the value of the oscillator strength of the exciton, as reported in Table S4. This procedure yields values of *N* ranging from 10 to 100, which are consistent with the literature.^20−22^ The excess of free molecules makes it difficult to retrieve the value of the UP and, in turn, of ℏΩ_R_. ℏΩ_R_ was thus calculated using the FDTD simulations of CPMs, where the UP maximum was clearly visible, and reported in Table S4. In conclusion, the model allowed us to estimate *W*_VAS_, which is strongly dependent on the *N* value. More in detail, exciting UP (ℏΩ_R_/(ℏν) = 1), *W*_VAS_ ∝ 1/*N*^2^, while exciting the DS (ℏΩ_R_/(ℏν) = 2), *W*_VAS_ ∝ 1/*N*. Consequently, we are expecting VAS to contribute more to the processes from the DS.

#### Surface-Enhanced Raman Scattering (SERS)

The SERS enhancement factor (EF_SERS_) quantifies the enhancement of the Raman signal due to SERS. This is one of the most important figures of merit for SERS and it is calculated as^30,65^
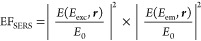
4where *E*_0_ is the *E*-field of the incident light, and *E*(*E*_exc_*,***r**) and *E*(*E*_em_*,***r**) are the *E*-fields of the CPM at the excitation and emission energies at coordinates ***r*** (Figure 1c). Although this model is quite simplistic for a complex system such as strongly coupled materials exciting in resonance, it has previously been used for calculating resonant SERS,^66,67^ and it qualitatively agrees with more advanced models.^68^

Previous works on plexcitonic materials reported that SERS can occur when exciting with laser sources the UP,^31,32^ the LP,^33−36^ or the DS.^33,37^ In this work, however, signals were detected only when exciting the UP and the DS, while no signal was observed when exciting the LP (Figure 4), most likely because of the weak excitation source used—a Xe lamp. When exciting the UP and when *ℏ*Ω_R_/(*ℏ*ν) = 1, both *E*(*E*_exc_*,***r**) and *E*(*E*_em_*,***r**) are in resonance with partially plasmonic states, thus providing a maximized EF_SERS_ for this condition. Instead, when the LP is excited, only *E*(*E*_exc_*,***r**) is in resonance with the LP, providing a smaller EF_SERS_, which only can be detected using laser excitation. The broad line shapes of UP and LP, and the partial bright content of the DS,^10,69,70^ results in an expected EF_SERS_, in-between that of when exciting the UP and LP, when exciting the DS. FDTD simulations (using an incident plane wave) were used to quantitatively evaluate EF_SERS_. For each CPM and region of emission, |*E*(*E*_exc_*,***r**)/*E*_0_|^2^ was calculated using the *E*_exc_ that yielded the maximum integrated emission. For the CPM reported in Figure 4c, for instance, the maximum emission for the 1410 cm^–1^ region was obtained exciting at 555 nm, so we used the corresponding energy to calculate |*E*(*E*_exc_*,***r**)/*E*_0_|^2^. |*E*(*E*_em_*,***r**)/*E*_0_|^2^ was calculated in the same way, using *E*_em_ = *E*_exc_ – *ℏ*ν.^71,72^

#### Comparison of VAS and SERS Models with the Experimental Values

**Figure 5:**
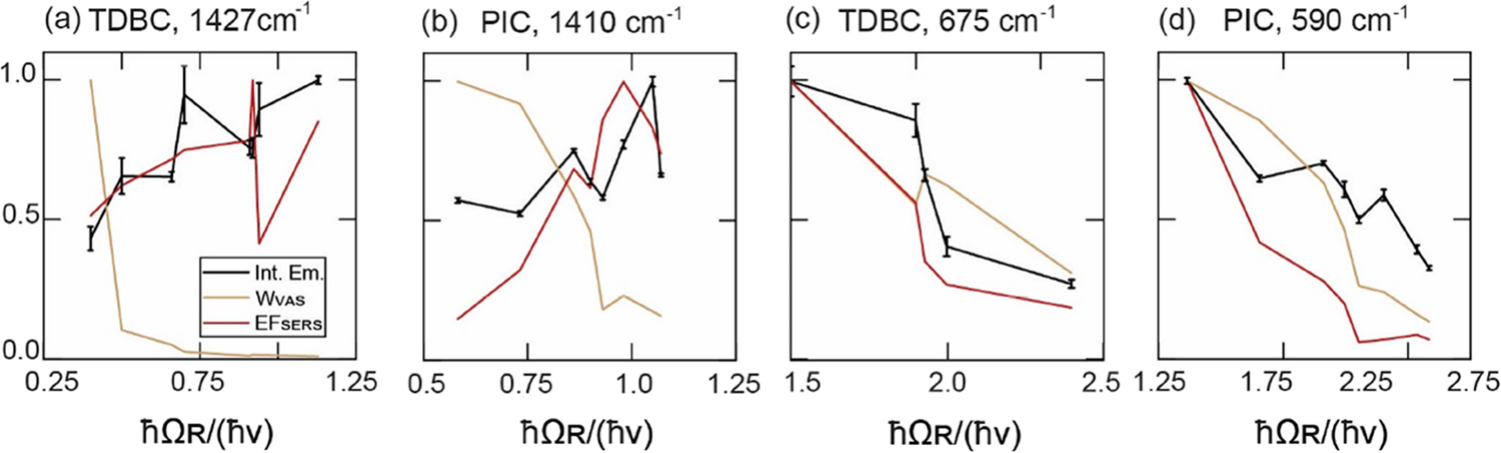
Comparison between the experimental emission of the CPMs and the calculated values of *W*_VAS_ and EF_SERS_. By varying the concentration of TDBC or PIC, TDBC- and PIC-CPMs were prepared with different *ℏ*Ω_R_. The CPMs were then classified by the *ℏ*Ω_R_ divided by the different emission regions *ℏ*ν (note that collected emission also contains scattering): (a) TDBC-CPMs, *ℏ*ν = 1427 cm^–1^, (b) PIC-CPMs, *ℏ*ν = 1410 cm^–1^, (c) TDBC, *ℏ*ν = 675 cm^–1^, and (d) PIC, *ℏ*ν = 590 cm^–1^. The black lines show the maxima of the integrated emissions in Figures 4c,d and S16b–S33b for every CPM reported. *W*_VAS_ and EF_SERS_ are values calculated for every CPM (see Section S5) using eqs 1 and 4, respectively. Light brown and red lines refer to *W*_VAS_ and EF_SERS_, respectively.

To compare the simulated VAS rates and the SERS enhancement factors with the experimental data, we cannot use the integrated emission retrieved at the same excitation wavelength, because the activation of the vibration via VAS or SERS depends on the positions of the UP and LP, which changes from CPM to CPM (see Figures 4c,d and S16–S33). Instead, the maxima of the integrated emission centered at 590 and 1410 cm^–1^ for PIC–CPMs and at 675 and 1427 cm^–1^ for TDBC-CPMs were considered (see also Section S4 in Supporting Info, and Figure S15). In total eight PIC-CPMs and eight TDBC–CPMs were analyzed (see Supporting Information, Section S7 for details). Figure 5 presents *W*_VAS_, EF_SERS_, and the experimental maxima of the integrated emission, each of them normalized and plotted as a function of *ℏ*Ω_R_/(*ℏ*ν). Since *W*_VAS_ and EF_SERS_ were not extrapolated from the emission data, they cannot be quantitatively compared. Moreover, *W*_VAS_ and EF_SERS_ have different physical meanings and units. Their relative weight on the overall integrated emission is challenging to estimate. However, the role of VAS and SERS can be deduced just by comparing their trends.

If the UP region is excited (*ℏ*Ω_R_/(*ℏ*ν) < 1.25; Figure 5a,b), EF_SERS_ is maximized when the Rabi splitting, *ℏ*Ω_R_, equals the vibrational quantum *ℏ*ν, i.e., *ℏ*Ω_R_/(*ℏ*ν) = 1 due to an increased plasmonic content in both *E*(*E*_exc_*,***r**) and *E*(*E*_em_*,***r**). In comparison, the calculated *W*_VAS_ are inversely proportional to *ℏ*Ω_R_/(*ℏ*ν). This is because *ℏ*Ω_R_ ∝ √*N*, while the *W*_VAS_ ∝ 1/*N*^*2*^. The experimentally observed emission from the region at 1427 cm^–1^ for CPMs prepared with TDBC (Figure 5a) is consistent with the expected trend of SERS rather than of VAS, indicating that the main mechanism is SERS. For the case of the CPMs prepared with PIC (Figure 5b), the emission is not dependent on *ℏ*Ω_R_/(*ℏ*ν), suggesting that both SERS and VAS mechanisms are active but at different *ℏ*Ω_R_/(*ℏ*ν). When exciting the UP and approaching *ℏ*Ω_R_ = *ℏ*ν, SERS is maximized and becomes the predominant mechanism. At the same time, VAS is strongly reduced due to the high *N* values required to satisfy the condition *ℏ*Ω_R_ = *ℏ*ν = 1400 cm^–1^. In contrast, VAS becomes more significant as *N* and *ℏ*Ω_R_ decrease, to the point where it dominates the emission for CPMs made with PIC below *ℏ*Ω_R_/(*ℏ*ν) = 0.75 (see Figure 5b). However, this does not apply to CPMs prepared with TDBC (see Figure 5a), most likely because TDBC requires three times the value of N compared to PIC to achieve the same *ℏ*Ω_R_ (Section S6 and Table S4).

When the DS region is excited (*ℏ*Ω_R_/(*ℏ*ν) > 1.5; Figure 5c,d), not only VAS but also SERS become inversely proportional to *ℏ*Ω_R_/(*ℏ*ν) because of the reduction of plasmonic content of the DS region. The emission from the CPMs follows the same trend as both mechanisms predict. Although discerning the active mechanism(s) directly is challenging, EF_SERS_ is 1 order of magnitude lower compared to *ℏ*Ω_R_/(*ℏ*ν) below 1.5 (Figure S37). Contrarily, *W*_VAS_ from the DS region is at least 1 order of magnitude higher (Figure S38) because it scales as 1/*N*, making it much faster than the *W*_*VAS*_ from the UP region, which scales as 1/*N*^*2*^. This suggests that VAS is the predominant mechanism when *ℏ*Ω_R_ is greater than *ℏ*ν.

#### Comparison between CPMs and Fabry–Pérot Cavities

Fabry–Pérot cavities coupled with TDBC J-aggregates (TDBC-FP, Figure S39a) were also prepared and analyzed (Section S8) to have a direct comparison with CPMs. The TDBC-FP was designed to have a ℏΩ_R_ of 1250 cm^–1^ (Figure S39b), which is similar to the ℏΩ_R_ of most TDBC-CPMs presented in our study (Table S4). The analysis of the emission of the TDBC-FP, collected with the same conditions as for the TDBC-CPMs, showed no significant VAS signals (Figure S40). This result was expected because the high *N* value in Fabry–Pérot cavities reduces the *W*_VAS_ (eqs 2 and 3). However, VAS was observed to occur from the DS, as already reported (Figure S41),^14,16^ only when a high-intensity laser source was used to excite TDBC-FP. Since, instead, in this study a weak Xe lamp was used as the excitation source, we can conclude that VAS signals are larger in strongly coupled plasmonic than Fabry–Pérot cavities.

## Conclusions

This study provides insights into the relaxation of plexcitonic excited states and aims to determine if the relaxation is similar to polaritonic materials based on Fabry-Pérot cavities. For the latter, the contributions from VAS and radiative pumping are fundamental to the overall dynamics. The relaxation mechanisms of plexcitonic materials were studied by analyzing the emission of 20 different CPMs, in which it was clear that VAS is active together with SERS. Surprisingly, these phenomena were observed using a weak light source, a Xe lamp, while usually lasers are used for detection. This suggests that CPMs enhance SERS more than weakly coupled plasmonic cavities. At the same time, we did not find evidence of VAS for TDBC-FP, suggesting that CPMs enhance VAS more than polaritonic materials prepared with Fabry–Pérot cavities. This is probably because the small *N* numbers in CPMs make the VAS rate much faster than other relaxation mechanisms. The combination of CPMs, which provide strong signals, and the use of a lamp, which is inherently broadband, might open a new way to approach sensing. Specifically, SERS is the predominant mechanism when exciting the UP in plexcitonic materials. This is because both the E-field for excitation and emission in SERS are enhanced by the UP and LP, respectively, when the Rabi splitting is equal to a vibrational quantum. Plexcitonic systems can therefore provide a platform for tuning the sensitivity to specific vibrations of SERS.

Fast relaxation toward the LP is a requisite for building low threshold polariton condensates, and VAS has been proposed to be an important mechanism to enhance it. In this work, we show that VAS is indeed the active relaxation mechanism in plexcitonic materials when exciting the DS. However, caution must be exercised when interpreting the emission of plexcitonic material because SERS can also be active in some cases.

## Experimental and Methods

### Synthesis of NRs

All reactants were purchased from Sigma-Aldrich and used without further purification. Milli-Q water was used in all experiments. NRs were prepared with a three-step Ag-assisted seeded growth,^47^ consisting of nucleation of small spherical nanoparticles (seeds), prereduction, and growth of NR. HAuCl_4_ (25 μL, 50 mM) was added to a CTAB solution (4.7 mL, 0.1 M). The temperature was set to 27 °C. The seeds were prepared by reducing the gold in solution with NaBH_4_ (0.3 mM, 10 mM). For the prereduction, 5-bromosalicylic acid (45 mg) and AgNO_3_ (480 μL, 10 mM) were added in this order to a CTAB solution (50 mL, 50 mM). The temperature was set at 30 °C. After 15 min, a HAuCl_4_ solution (0.5 mL, 50 mM) was added under stirring. The prereduction reaction in solution was monitored by measuring the absorbance of the AuBr_4_^–^ complex, which exhibits a peak at 396 nm. When this peak reached values of absorbance equal to 0.74, ascorbic acid (130 μL, 100 mM) and gold seeds (80 μL) were added to the solution in this order. The reaction was left undisturbed for 4 h at 30 °C. Excess reactants and undesired byproducts were removed with two centrifugation steps (30 min, 6080 rcf). After each centrifugation step, the supernatant was removed, and the pellet was redispersed in Milli-Q water. The NRs were characterized by a longitudinal plasmon at 793 nm (see Figure S1). TEM measurements revealed a height of 53 ± 5 nm and a length of 14.20 ± 1.88 nm (Figure S2).

### Partial Oxidation of NR

For the partial oxidation of gold NRs, a procedure proposed by Rodriguez-Fernandez et al. was followed.^48^ For the CPMs presented in the main text, 1.322 mL of Au^3+^-CTAB complex ([Au] = 1, [CTAB] = 100 mM) was added dropwise under magnetic stirring to a vial containing 7.686 mL of the NR ([Au^0^] = 1, [CTAB] = 100 mM). The solution was allowed to react at 30 °C for 1 h. Subsequently, excess gold salt and byproducts were removed by centrifugation (3500 rcf, 30 min) and redispersed in Milli-Q water. Figure S3 shows TEM images and histograms of partially oxidized NR. The concentration of bulk gold in solution (Au^0^) can be estimated from the absorption of the nanoparticles.^47^ An absorption value of 1.2 at 400 nm, with an optical path of 1 cm, corresponds to [Au^0^] = 0.5 mM. Assuming that no other particles are present in the solution and considering the height and diameter of the NR 24.2 ± 5.3, and 14.5 ± 1.9 nm, respectively, an [Au^0^] = 0.5 mM corresponds to a [NR] = 0.88 nM.

### Preparation of NR@PSS

The PSS layer was added following a procedure reported in the literature by Ni et al.^73^ Briefly, 2 mL of a 0.51 nM NR solution was prepared and added dropwise to a 2 mL PSS solution (MW = 70,000, 6 mM NaCl) under vigorous stirring. The concentration of PSS was tuned from 1.7 to 2.6 mg/mL according to the desired capping layer thickness. The solution was left stirring at 30° for 3 h, centrifuged at 3500 rcf for 30 min, and redispersed in Milli-Q water.

### Preparation of CPMs

A 0.5 mM PIC solution in ethanol (0.25 to 6 μL) was added to 400 μL of a 0.286 nM NR@PSS solution and left undisturbed overnight. The volume of the added TDBC solution was between 16 and 110 μL. The extinction spectra of the dispersions of the CPMs were measured the day after. The CPMs with a large excess of free molecules were centrifuged (4000 rcf, 20 min) and redispersed in the same amount of Milli-Q water (see Table S1).

### TEM Measurements and Staining Procedure

TEM measurements were performed using a FEI Tecnai G2 Spirit BioTWIN (Thermofisher Scientific), operated at 120 kV and equipped with a FEI Ceta 16 M (4k × 4k) camera (Thermofisher Scientific). For the staining procedure, 5 μL of CPMs was applied to a glow-discharged carbon-coated Formvar 200-mesh grid and incubated for 10 min. Grids were washed twice with phosphate saline buffer and then fixed using 2.5% glutaraldehyde for 5 min. They were then washed with filtered distilled water and stained using 2% uranyl acetate in water for 1 min.

### Zeta Potential Measurements

Zeta potential measurements were performed using a Zetasizer Nano Z (Malvern Panalytical Ltd., Malvern, UK). The instrument was equipped with a 633 nm laser. The temperature was set at 25 °C. The data were analyzed with the Zetasizer software.

### Optical Measurements

Absorption and extinction spectra were measured using a standard spectrophotometer (Lambda 950, PerkinElmer). Emission measurements were performed using an FLS 1000 spectrofluorometer (Edinburgh Instruments). Each CPM prepared with PIC was excited from 505 to 610 nm with a step of 5 nm. CPMs prepared with TDBC presented stronger background emission, consequently, the excitation was collected from 540 to 610 nm, with a step of 5 nm. The emission was scanned up to 690 nm, with a step of 1 nm and an integration time of 1 s. The scan and the detector slits were set at 2 and 3 nm, respectively. The error bars reported in Figures 4c,d and 5 were calculated for every λ_exc_ using the noise (the standard deviation of the signal) in the region between 1750 and 2500 cm^–1^. The noise σ_noise_ can then be considered as the error for every emission wavelength. The error bar for the integrated emission σ_int_ can be calculated from the general error propagation formula as σ_int_ = σ_noise_ √(Int. points), where “Int. points” are the number of integrated wavelengths used for the integrations. Raman measurements were carried out using a confocal Raman microscopy setup (WITec alpha300R, grating 600 mm^–1^, spectral resolution 3 cm^–1^, continuous wave laser excitation 488 nm, objective Zeiss EC Epiplan-Neofluar Dic 100*x*/0.9).

### FDTD Simulations

FDTD simulations were performed using the Lumerical FDTD software. The best agreement with the experimental measurements was achieved by simulating the NR as a cylinder with two hemispheres placed on the top and bottom bases. The total diameter and height of the simulated NR were 15.5 and 24 nm, respectively. The PSS thickness was set at 1 and 2.25 nm for rods with and without PIC (or TDBC), respectively. These values were based on TEM measurements (Figures 1c,f, S5, and S6). The refractive index of water was set to 1.33. The dielectric permittivity ε(ω) of the gold core of the NR was taken from literature.^74^ The dielectric permittivity of the capping layer was modeled using a Lorentzian function:
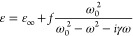
5where ω_0_ is the absorption maximum, *f* is the oscillator strength, and ε_∞_ is the high-frequency dielectric constant of the dye aggregate. ω_0_ was set to 17,094 cm^–1^ and γ to 241 cm^–1^, for both J-aggregates excitons, as retrieved from the experimental data. *f* and ε_*∞*_ were varied depending on the molecule and their concentration, as reported in Tables S2 and S3. All simulations were carried out with the NRs in water. The mesh sizes on the region of the capping layer were set to 0.25 nm for the simulation of the extinction and 0.125 for the E-field. This selection was based on prior testing for numerical convergence.

## Supporting Information Available

Synthesis and characterization of NR; characterization of NR@PSS with different PSS loading; coupling NR@PSS with PIC and TDBC J-aggregates; additional emission spectra of CPMs; control emission experiment with PIC/TDBC bound to long NR@PSS; further information about the calculation of W_VAS_; further information on the comparison between EF_SERS_, *W*_VAS_, and experimental emission; preparation and characterization of the emission of Fabry-Pérot cavities coupled with TDBC J-aggregates (TDBC-FP); and raw data is stored at the Swedish National Data Service with the digital object identifier 10.5878/ekr1-v904 (10.5878/ekr1-v904) (PDF)

## Abbreviations

CPMs: colloidal plexcitonic materials
CTAB: cetyltrimethylammonium bromide
DS: dark states
E-field: electromagnetic field
EF_SERS_: SERS enhancement factor
FDTD: finite-difference time-domain
LP: lower plexciton/polariton
NRs: gold nanorods
NR@PSS: NRs coated with PSS
PSS: polystyrenesulfonate
SERS: surface enhanced Raman scattering
TDBC-FP: Fabry-Pérot cavities coupled with TDBC J-aggregates
VAS: vibrational-assisted scattering
ℏΩ_R_: Rabi splitting
UP: upper polariton/plexciton
