# Supplementary material for: Role of Vibrational-Assisted Scattering and Surface-Enhanced Raman Scattering in Colloidal Plexcitonic Materials
Source: ACS Nano. 2025 Apr 16;19(16):15627–37. doi: 10.1021/acsnano.4c17571 (PMC12044703; doi:10.1021/acsnano.4c17571)
Supplement: Supplementary file 1 — nn4c17571_si_001.pdf [file nn4c17571_si_001.pdf]

The Role of Vibrational-Assisted Scattering and Surface-Enhanced Raman Scattering in Colloidal Plexcitonic Materials

Supporting Information

Nicola Peruffo,[1] Minpeng Liang,[2] Rahul Bhuyan,[1] Vajradhar Acharya,[1] Johanna L. Höög,[1] Karl Börjesson[1]

[1] Department of Chemistry and Molecular Biology, University of Gothenburg, 41390 Göteborg, Sweden

[2] Department of Applied Physics and Science Education, Eindhoven Hendrik Casimir Institute and Institute for Complex Molecular Systems, Eindhoven University of Technology, 5612AE Eindhoven, The Netherlands

Index

1. Characterization of NR 3
2. Loading of NR with PSS to form NR@PSS 6
3. Coupling NR@PSS with PIC and TDBC J-aggregates 8
4. Additional emission spectra of CPMs 17
5. Control emission experiment with PIC/TDBC bound to long NR@PSS 30
6. Further information about the calculation of the W_{VAS} 32
7. Further information on the comparison between emission, EF_{SERS}, W_{VAS}, and the emission 34
8. Preparation and characterization of the emission of Fabry-Pérot cavities coupled with TDBC J-aggregates (TDBC-FP) 38
9. References 44

1. Characterization of NR

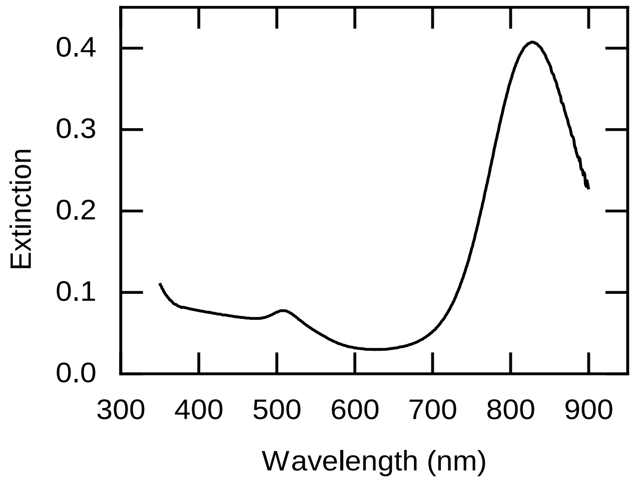

Figure S1: Extinction of NRs before partial oxidation.

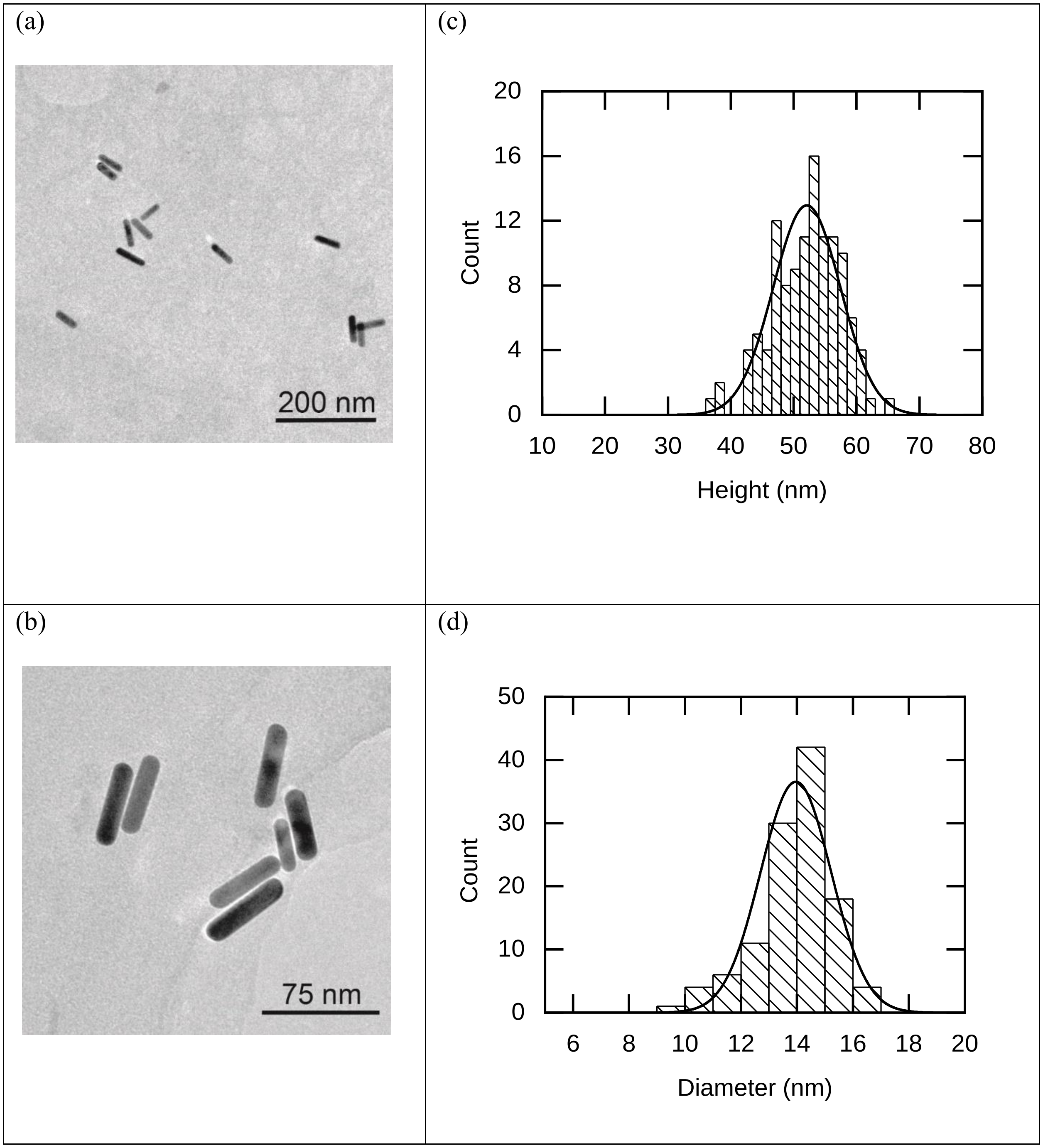

Figure S2: TEM analysis of NR before oxidation. a) and b): TEM micrographs at different scales; c) the histogram of the distribution of the heights and d) of the diameters of 200 measured NRs.

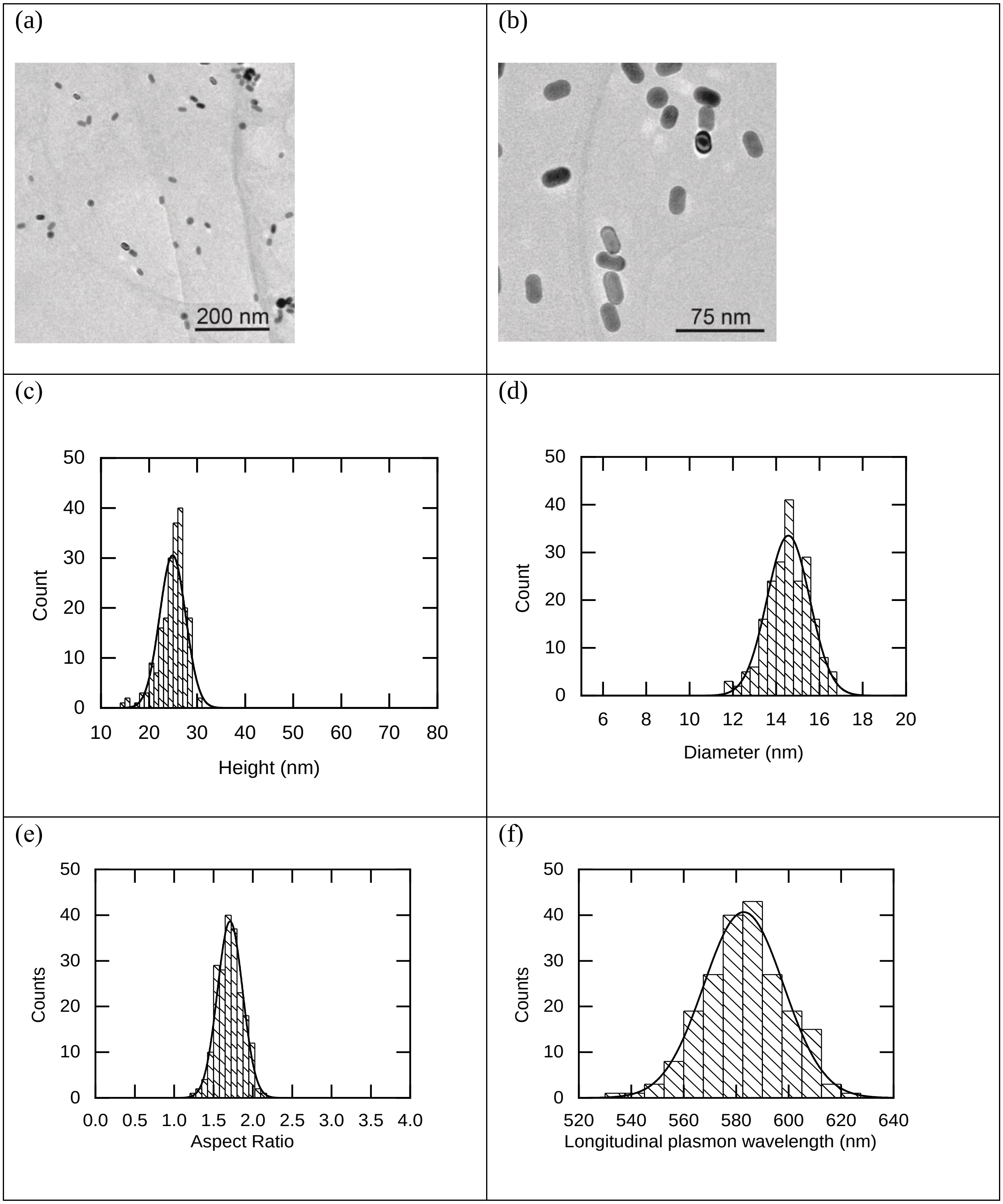

Figure S3: TEM analysis of NRs after oxidation. a) and b): TEM micrographs at different scales; c) histogram of the distribution of the heights, d) of the diameters and e) of the aspect ratio of 200 measured NRs. f) distribution of the wavelengths of the longitudinal plasmon, calculated from the aspect ratio.

2. Loading of NR with PSS to form NR@PSS

The thickness of the PSS layer was adjusted by varying the amount of PSS added to the NRs solution. In this Section, NR@PSS prepared with 1.7 mg/mL and reported in the main text are characterized and compared to NR@PSS prepared with 2.0 mg/mL of PSS. The zeta potential of NR was measured to be -24 and -33 meV when adding 1.7 and 2.0 mg/mL of PSS, respectively. The extinction of the longitudinal plasmon shifted from 578 nm to 572 nm for NR loaded with 1.7 mg/mL of PSS, and to 571 nm for NR loaded with 2.0 mg/mL of PSS (Figure S4). The two NR@PSS have also different layer thicknesses, being 1.3 ± 0.2 nm and 1.7 ± 0.4 nm for NR@PSS with 1.7 mg/mL and 2.0 mg/mL, respectively (Figure S5). This analysis confirms that the PSS thickness on the NR surface increases with the amount of PSS added to the NR solution.

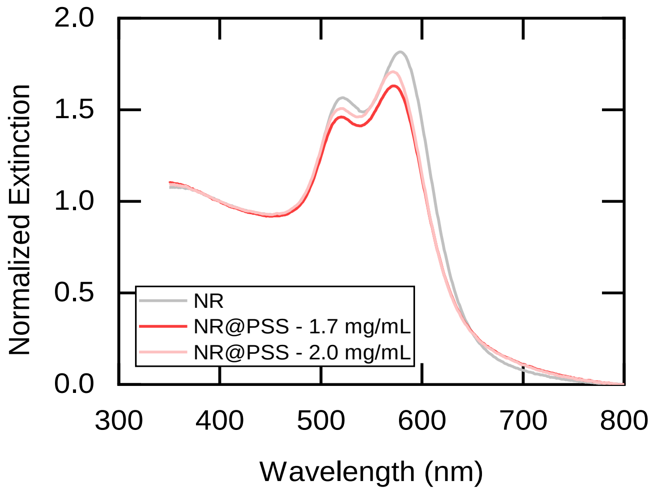

Figure S4: Normalized extinction of NR and NR@PSS adding 1.7 and 2.0 mg/mL of PSS. The NR extinction spectra are normalized at 400 nm.

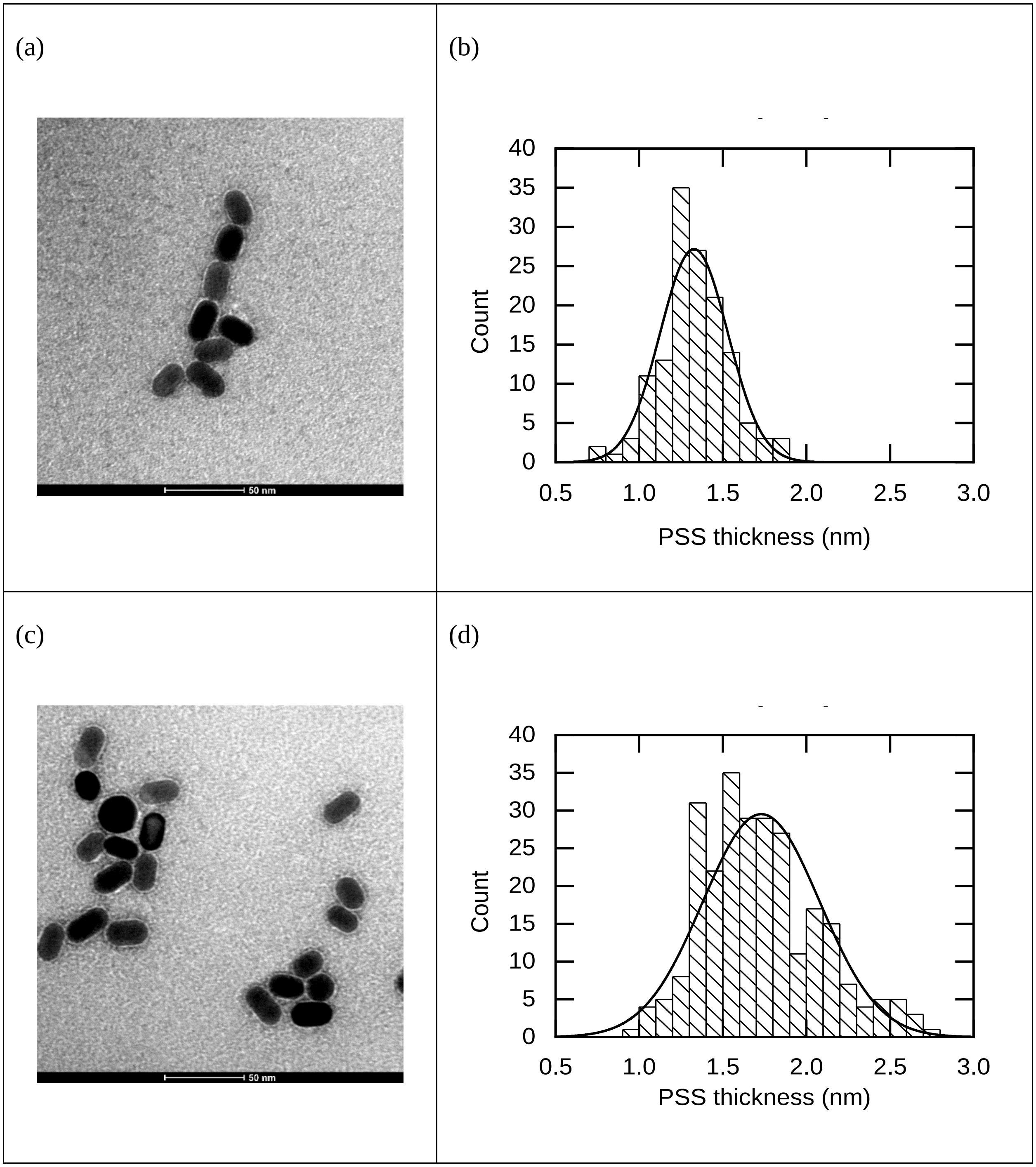

Figure S5: a) TEM micrographs of NR@PSS when adding a 1.7 mg/mL PSS solution; b) histogram of the relative PSS thickness distribution over 100 measures. The same analysis is reported in panels c) and d) for NR@PSS prepared with 2.0 mg/mL of PSS.

3. Coupling NR@PSS with PIC and TDBC J-aggregates

The binding between NR@PSS and PIC resulted in a capping layer of 1.9 ± 0.8 nm. The histogram of the distribution of the thicknesses is reported in Figure S6. The strong coupling between NR@PSS and PIC/TDBC J-aggregates was evaluated in 20 CPMs prepared with 3 different NR batches, 5 PSS loadings, and several dye concentrations. The code and composition of each CPM was reported in Table S1. The extinction spectra of the CPMs are reported in Figures S7-S11. Each panel of Figures S7-S11 reports a different NR@PSS and all the CPMs prepared with it.

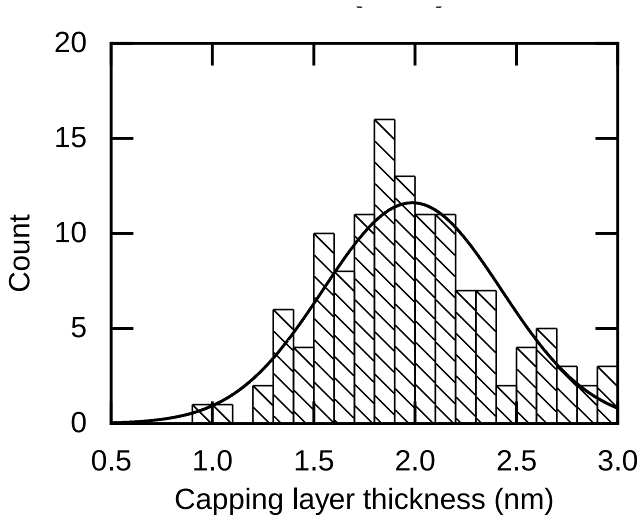

Figure S6: Histogram of the distribution of the thickness of the NR@PSS coupled with PIC.

Table S1: For each CPM, a code is assigned. The columns report the CPM code, the NR batch, the PSS loading. The final column reports the dye, its concentration in the CPMs solution, and whether it has been washed to remove the excess of dye.

CPM code	NR batch	PSS loading (mg/mL)	Dye - concentration (µM)
**1**	1	1.7	PIC – 0.3
**2**	1	1.7	PIC – 0.6
**3**	1	1.7	PIC – 7.4
**4**	1	1.7	PIC – 7.4 - washed
**5**	1	2.0	PIC – 0.3
**6**	1	2.0	PIC – 0.6
**7**	1	2.0	PIC – 7.4
**8**	1	2.0	PIC – 7.4 – washed
**9**	2	2.0	PIC – 7.4 – washed
**10**	2	2.6	PIC – 7.4 – washed
**11**	3	2.0	PIC – 7.4 – washed
**12**	1	1.7	TDBC – 19
**13**	1	1.7	TDBC – 24
**14**	1	1.7	TDBC – 28 - washed
**15**	1	2.0	TDBC – 19
**16**	1	2.0	TDBC – 24
**17**	1	2.0	TDBC – 28
**18**	1	2.0	TDBC – 154 - washed
**19**	1	2.3	TDBC – 154 - washed
**20**	2	2.0	TDBC – 154 - washed

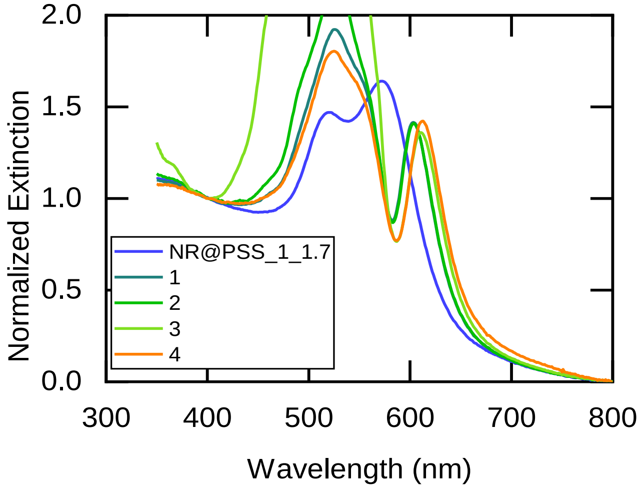

Figure S7: Normalized extinction of CPMs 1-4, compared with the respective NR@PSS batch (NR batch 1, PSS loading 1.7 mg/mL). The extinction spectra are normalized at 400 nm.

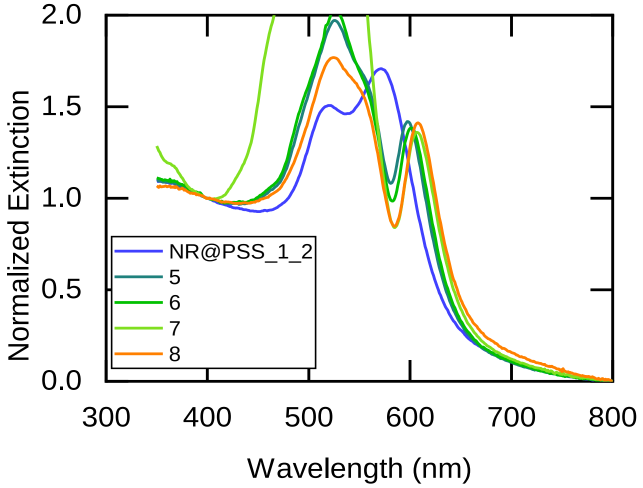

Figure S8: Normalized extinction of CPMs 5 – 8, compared with the respective NR@PSS batch (NR batch 1, PSS loading 2 mg/mL). The extinction spectra are normalized at 400 nm.

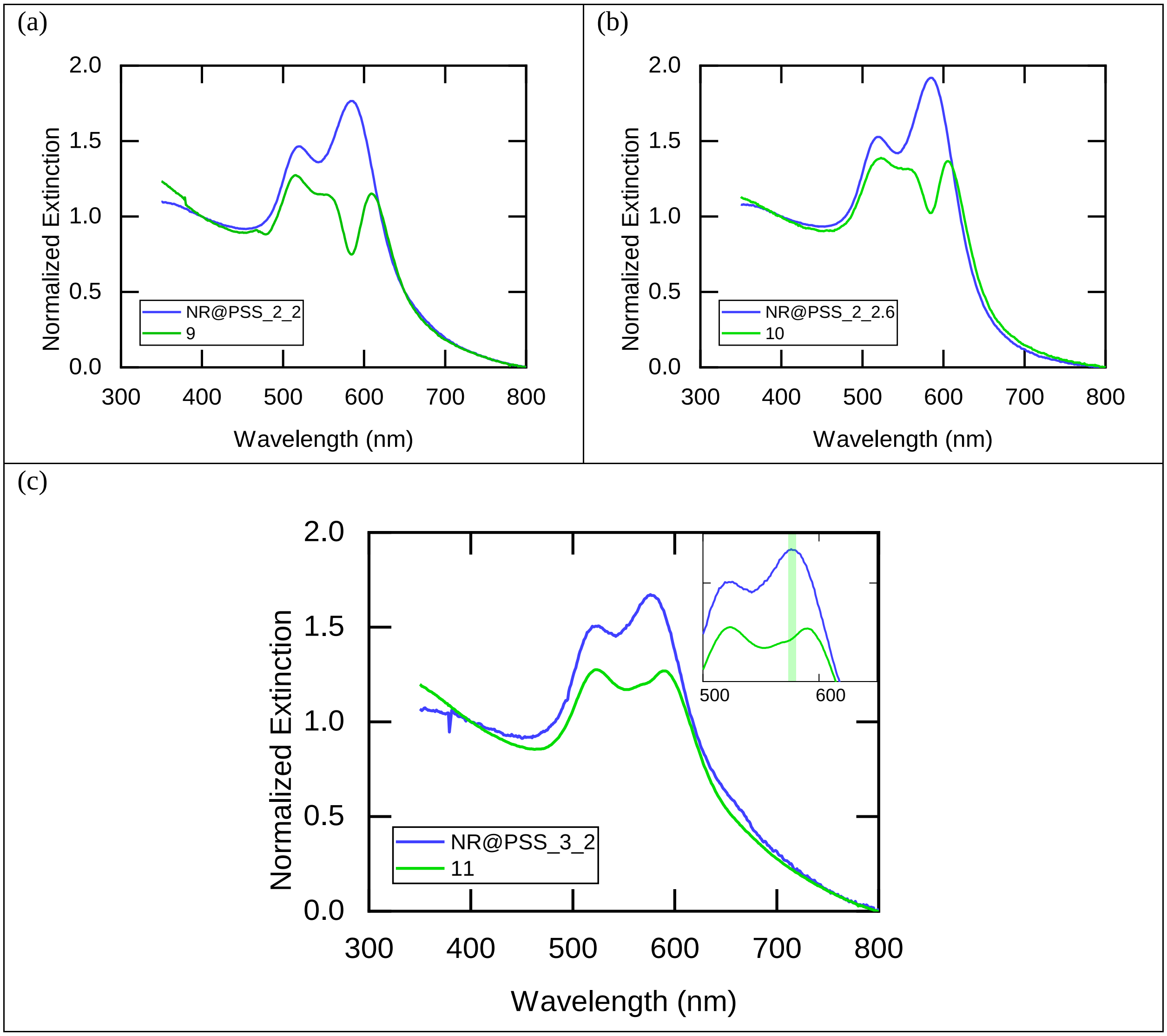

Figure S9: a) Normalized extinction of CPMs 9, b) 10, c) 11 compared with the respective NR@PSS batches ((NR batch 2, PSS loading 2 mg/mL for (a); NR batch 2, PSS loading 2.6 mg/mL for (b); NR batch 3, PSS loading 2 mg/mL for (c)). An inset in panel (c) shows the modest $\hbar\Omega_R$ of CPM 11. The green line represents the position of the exciton of PIC J-aggregates. The extinction spectra are normalized at 400 nm.

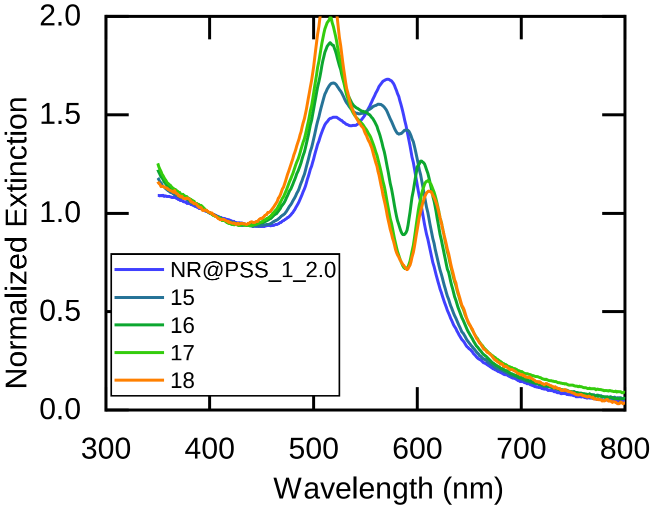

Figure S10: Normalized extinction of CPMs 15 – 18, compared with the respective NR@PSS batch (NR batch 1, PSS loading 2 mg/mL). The extinction spectra are normalized at 400 nm.

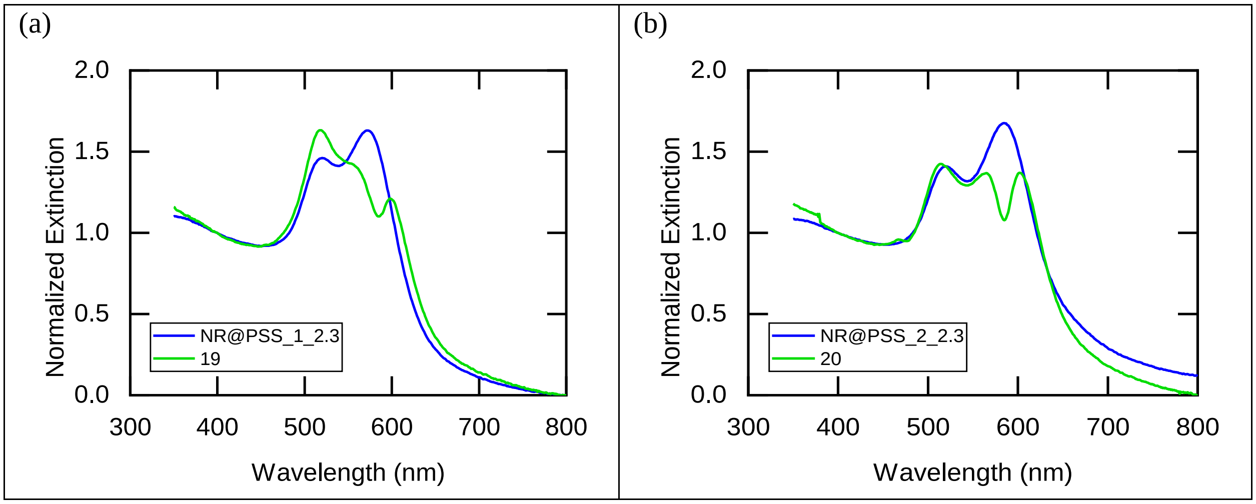

Figure S11: a) Normalized extinction of CPMs 19, and b) 20 compared with the respective NR@PSS batches (NR batch 1, PSS loading 2.3 mg/mL for (a); NR batch 2, PSS loading 2.3 mg/mL for (b)). The extinction spectra are normalized at 400 nm.

CPMs 1-8 and 12-19 were simulated and reported in Figures 3, S12 and S13. In FDTD simulations, the dielectric permittivity of the capping layer was modelled using a Lorentzian function:

$$\varepsilon = \varepsilon_{\infty} + f\frac{\omega_0^2}{\omega_0^2 - \omega^2 - i\gamma\omega}$$

where ω_0 is the absorption maximum, f is the Lorentzian oscillator strength, and ε_∞ is the high-frequency dielectric constant of the dye aggregate. ω_0 was set to 17390 and 16980 cm^{-1} for PIC and TDBC, respectively. The absorption peak of both dyes reported a half-width at half-maximum equal to 242 cm^{-1}, used as $\hbar\gamma$. f and ε_∞ were varied to match the experimentally observed Rabi splitting. They keep in account that an increase of PIC/TDBC in the capping layer changes the ε_∞ and increases f. The values are reported in Table S2 and S3.

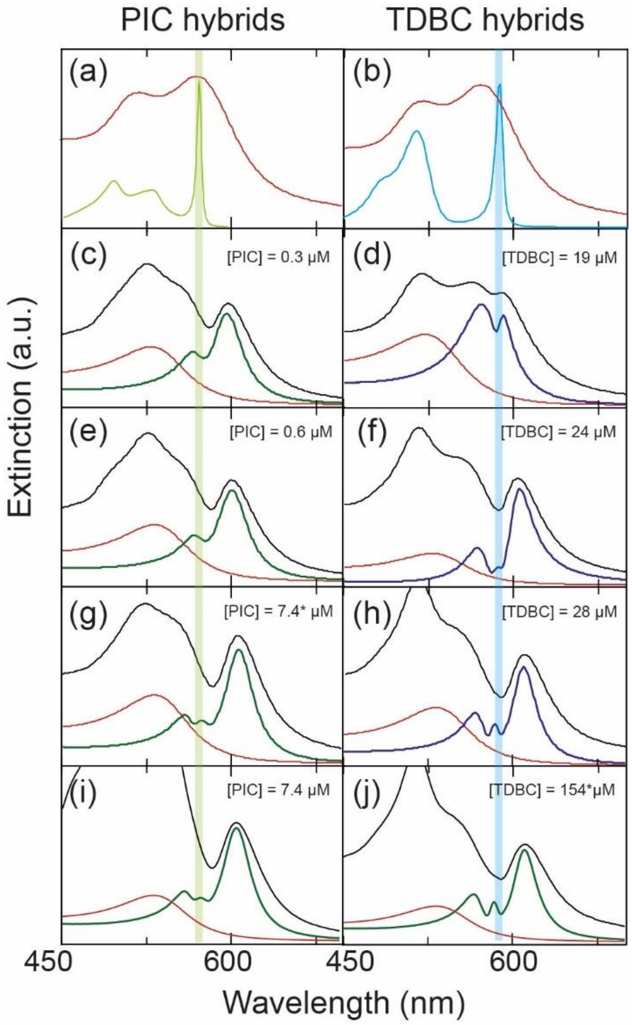

Figure S12: (a) Experimental extinction spectrum of the NR@PSS (red line) and experimental absorption of the J-aggregate of PIC (green line). (b) Experimental extinction spectrum of the NR@PSS (red line) and experimental absorption of the J-aggregate of TDBC (cyan line). In all the panels, a colored vertical line signals the excitonic wavelength of the J-aggregates. (c) Experimental extinction of CPMs prepared with [PIC] = 0.3 µM (black line), and simulated extinction with polarization along the short (dark red) and long (dark green) axes. The same spectra are reported in (e), (g), and (i) with increasing [PIC] from 0.6 µM to 7.4 µM. (d) experimental extinction of CPMs prepared with [TDBC] = 0.3 µM (black line), and simulated extinction with polarization along the short (dark red) and long axes (dark blue). The same spectra are reported in (f), (h), and (j), with increasing [TDBC] from 24 µM to 154 µM. For CPMs in panels (g) and (j), the extinction is reported after a washing step. The Figure is prepared in the same fashion as Figure 3, to ease the comparison. All the CPMs were prepared with a PSS loading of 2 mg/mL.

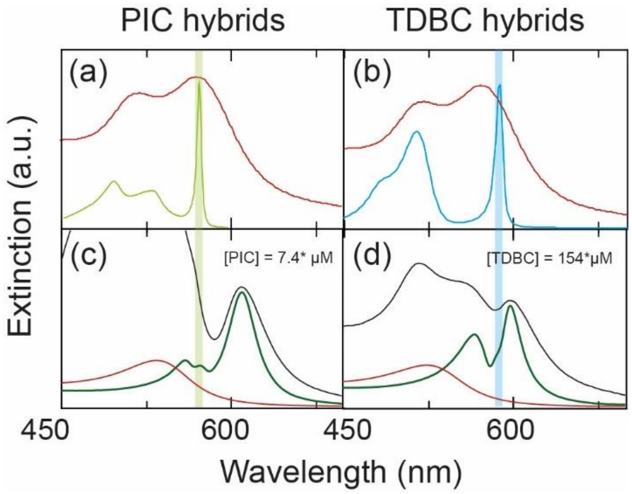

Figure S13: (a) Experimental extinction spectrum of the NR@PSS (red line) and experimental absorption of the J-aggregate of PIC (green line). (b) Experimental extinction spectrum of the NR@PSS (red line) and experimental absorption of the J-aggregate of TDBC (cyan line). In all the panels, a colored vertical line signals the excitonic wavelength of the J-aggregates. (c) Experimental extinction of CPM 3 (black line; reported after a washing step) and simulated extinction with polarization along the short (dark red) and long (dark green) axes. (d) Experimental extinction of CPM 19 (black line; reported after a washing step), and simulated extinction with polarization along the short (dark red) and long (dark blue) axes. The Figure is prepared in the same fashion as Figure 3, to ease the comparison.

Table S2: f and ε_∞ parameters used in the FDTD simulations of PIC-based CPMs.

CPM code	ε_∞	f
1	3	0.0475
2	3	0.055
3	3.3	0.095
4	3.3	0.1
5	2.65	0.03
6	3	0.035
7	3	0.0825
8	3	0.09

Table S3: f and ε_∞ parameters used in the FDTD simulations of TDBC-based CPMs.

CPM code	ε_∞	f
12	2.25	0.02
13	3	0.075
14	3	0.1
15	2	0.006
16	2.6	0.05
17	3	0.08
18	3	0.09
19	2.25	0.035

4. Additional emission spectra of CPMs

Figure 4 in the main text reported a detailed analysis of the emission of CPMs 1 and 12. In this Section, the analysis is expanded to the other 18 CPMs classified in Table S1. The CPMs prepared with PIC were excited from 505 nm to 610 nm every 5 nm, while the excitation for CPMs prepared with TDBC starts at 540 nm, because of the high amount of emission from free molecules. A comparison between the emission of a PIC (TDBC) solution and the corresponding PIC-CPM (TDBC-CPM) is shown in Figure S14, highlighting that a background emission exists for all excitation wavelengths and that no SERS/VAS-like signals are present for PIC/TDBC in solution.

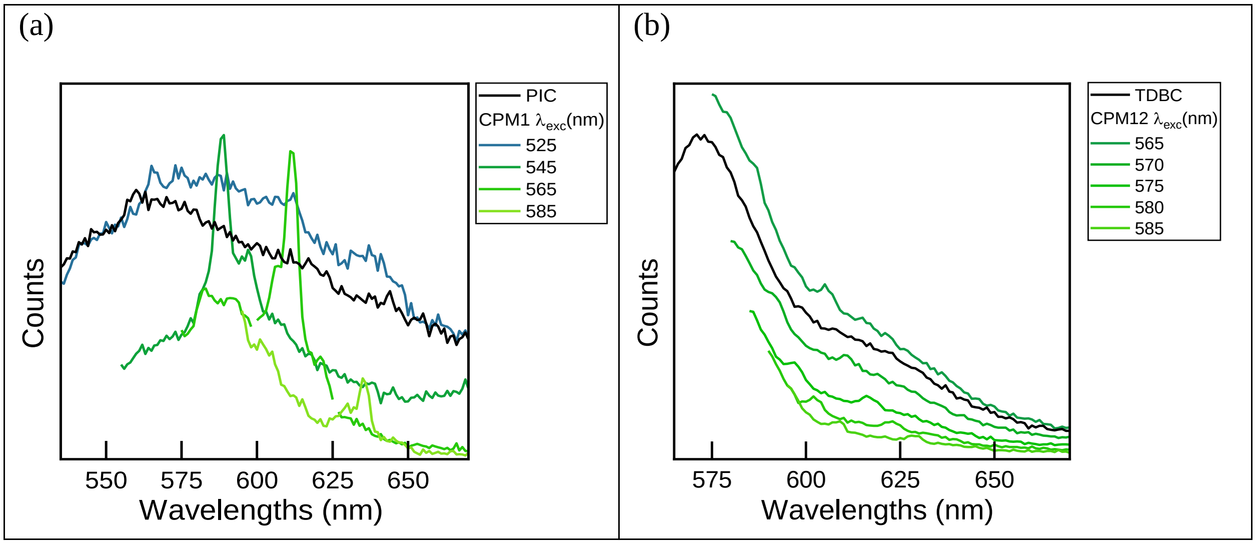

Figure S14: a) Emission of a PIC solution (1 μM, λ_{exc} = 525 nm, black curve), compared with the emission at different λ_{exc} of CPM 1. b) Emission of a TDBC solution (1 μM, λ_{exc} = 555 nm, black curve), compared with the emission of CPM 12 excited at different wavelengths.

Figure S15 shows an overview of all the mathematical operations employed to plot Figures 4, 5 and S16 to S33 using as an example CPM1. The emission was plotted in function of λ_{exc} to distinguish better the background emission (Figure S15a). The background was removed using a background removing function of the software Origin 2018© (Figure S15b). The remaining

emission was plotted as a function of the Raman shift and divided into regions corresponding to the vibrational peaks of PIC/TDBC. Figure S15c shows the emission spectrum while exciting at 555 nm, and the integrated spectrum is shown as a green area. Figure S15d shows the integrated emissions as a function of the excitation wavelengths. For each emission region, a clear maximum with respect to excitation wavelength can be seen. The point of maximum integrated emission corresponds to the excitation wavelength of 555 nm, thus the spectrum shown in Figure 15c (as shown by the green arrow). In Figure 5, the maximum of the integrated emission for each emission region ħν and for each CPM is reported. In this examples, CPM1 has $\hbar\Omega_R/(\hbar\nu) = 0.86$. Since exciting at 555 nm resulted in the maximum of the integrated emission in the vibrational region 1410 cm^{-1}, that value is used to compose one point in Figure 5b, as visually represented with a black arrow from Figure S15d to Figure S15e.

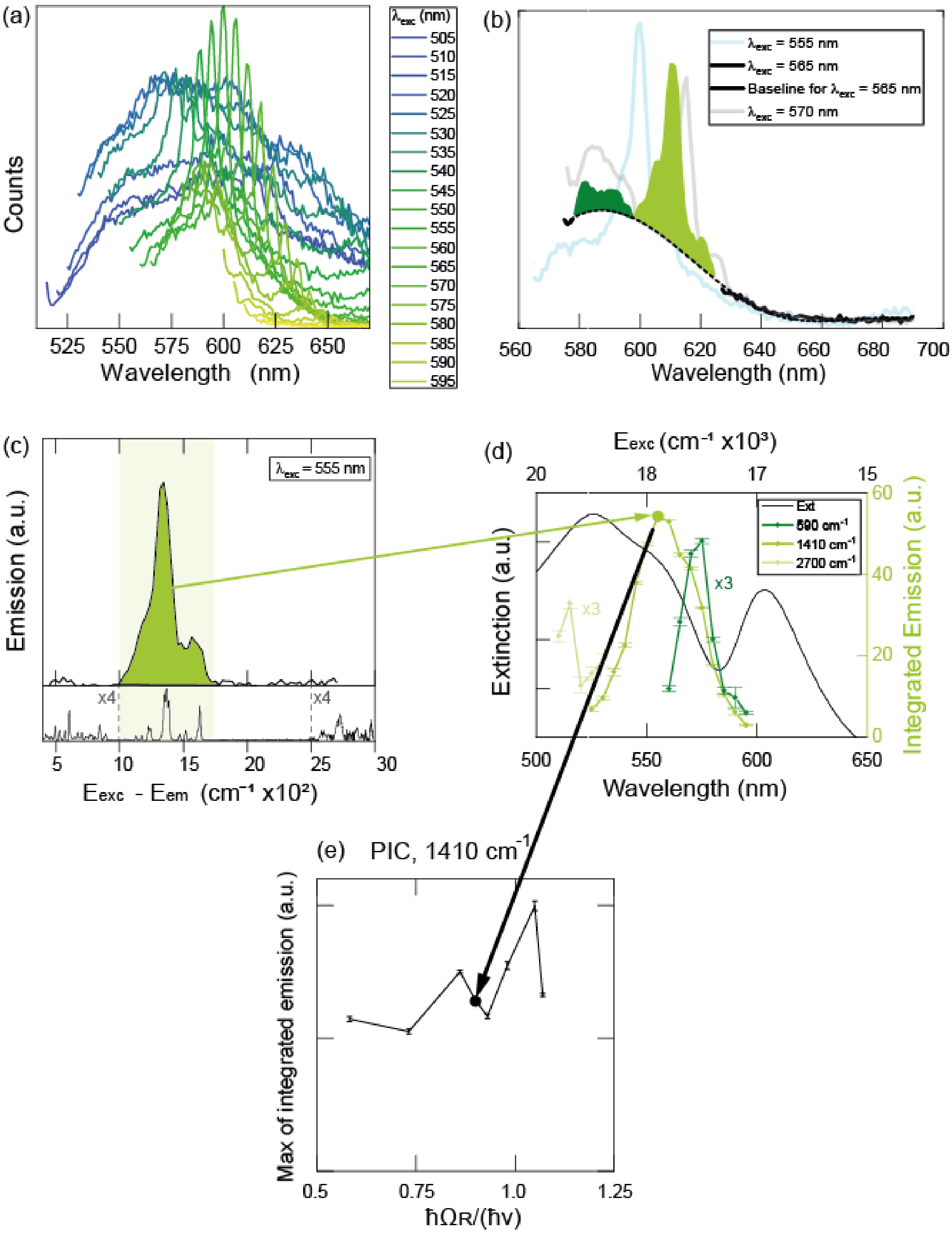

Figure S15: a) Raw emission spectrum of CPM1 in function of the wavelength. b) Emission spectra when exciting at λ_{exc} = 565, 570 and 575 nm. In the graph, the background emission for λ_{exc} = 565 is removed (dotted lines). c) Emission spectrum λ_{exc} = 555 nm free of background. The remaining emission is mainly coming around the energy of the vibration $\hbar\nu$ = 1410 cm^{-1}. The area of the integrated emission is shown in green. d) Integrated emission at all the excitation wavelengths and at different vibrational regions. The extinction of CPM1 is reported as a black line as well. e) Since the integrated value of the emission at 555 nm and for the vibrational region of 1410 cm^{-1} is the highest, it is used as one point of the emission curve of Figure 5b, as graphically reported here.

The raw emission of the CPMs (Figure S16 – S33) is reported as a function of $E_{exc} – E_{em}$. The emission was measured exciting from 505 nm to 610 nm for PIC, and from 540 nm to 610 nm, every 5 nm. However, because of the high levels of background emission in some CPMs, the emissions retrieved at lower excitation wavelengths were not always plotted (see captions of Figure S16 – S33 for more information). To extract the VAS/SERS signals, the background emission was subsequently subtracted using the method displayed in Figure S15. The patterns reported are similar to those in the main text. The integration of the emission along the main vibrations are in agreement with those reported in the main text too. CPM 11 (Figure S9a and S25) possesses the lowest Rabi splitting and the lowest emission among the PIC-CPMs: below this coupling, we are in the weak coupling regime and therefore do not expect VAS/SERS signals.

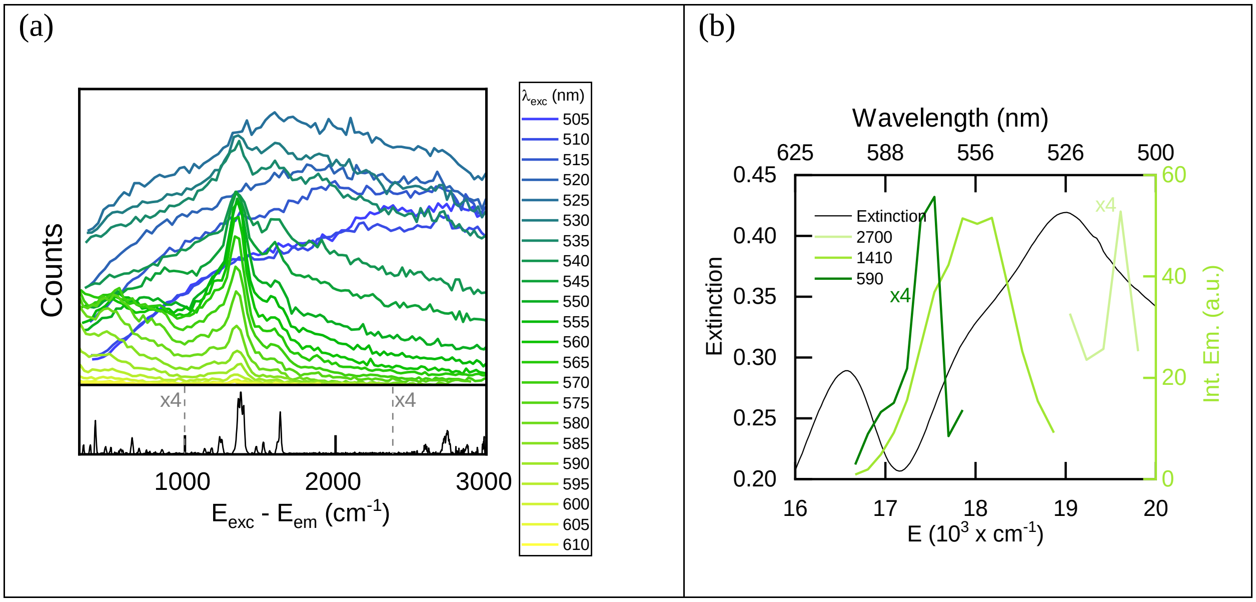

Figure S16: a) Raw emission spectra (background + VAS/SERS) in function of E_{exc}-E_{em} of CPM 2. The Raman spectrum of PIC is plotted below, with the regions below 1000 cm^{-1} and above 2500 cm^{-1} scaled by a factor x4. The emission is plotted from λ_{exc} = 505 nm (blue line) to 610 nm (yellow line) every 5 nm. b) Integrated emission of the signals from the region centered at 590 cm^{-1} (dark green curve, scaled by a factor of 4), 1410 cm^{-1} (green curve), and 2700 cm^{-1} (light green curve) in function of the excitation wavelength. The extinction spectrum of CPM 2 is superposed and the scalebar reported on the left.

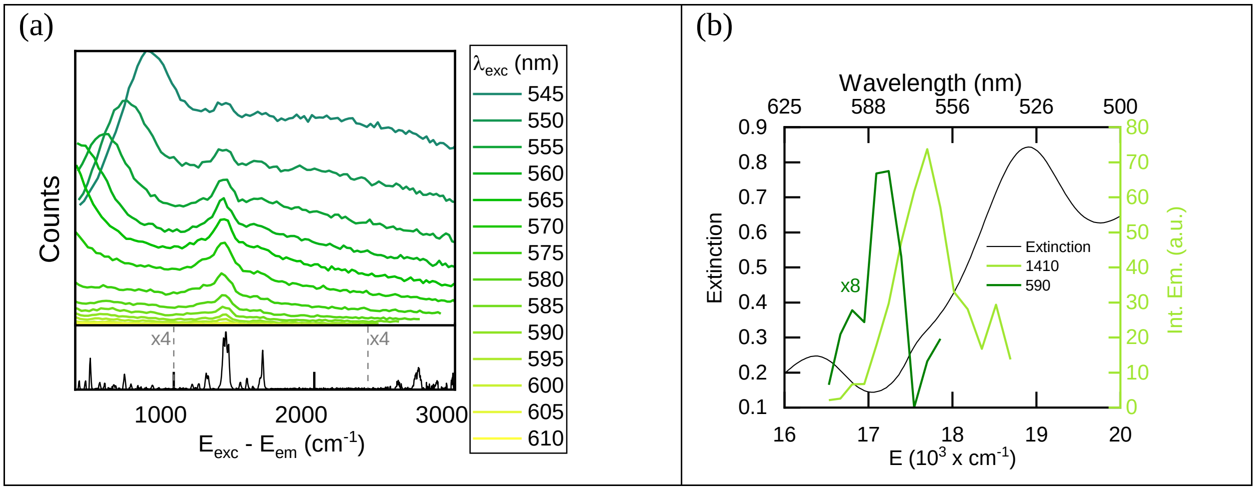

Figure S17: a) Raw emission spectra (background + VAS/SERS) in function of E_{exc}-E_{em} of CPM 3. The emission is plotted from λ_{exc} = 545 nm (green line) to 610 nm (yellow line) every 5 nm. The Raman spectrum of PIC is plotted below, with the regions below 1000 cm^{-1} and above 2500 cm^{-1} scaled by a factor x4. The signal in the region centered at 590 cm^{-1} is barely recognizable for this CPM. b) integrated emission of the signals from the region centered at 590 cm^{-1} (dark green curve, scaled by a factor of 8), and 1410 cm^{-1} (green curve). The extinction spectrum of CPM 3 is superposed and the scalebar reported on the left.

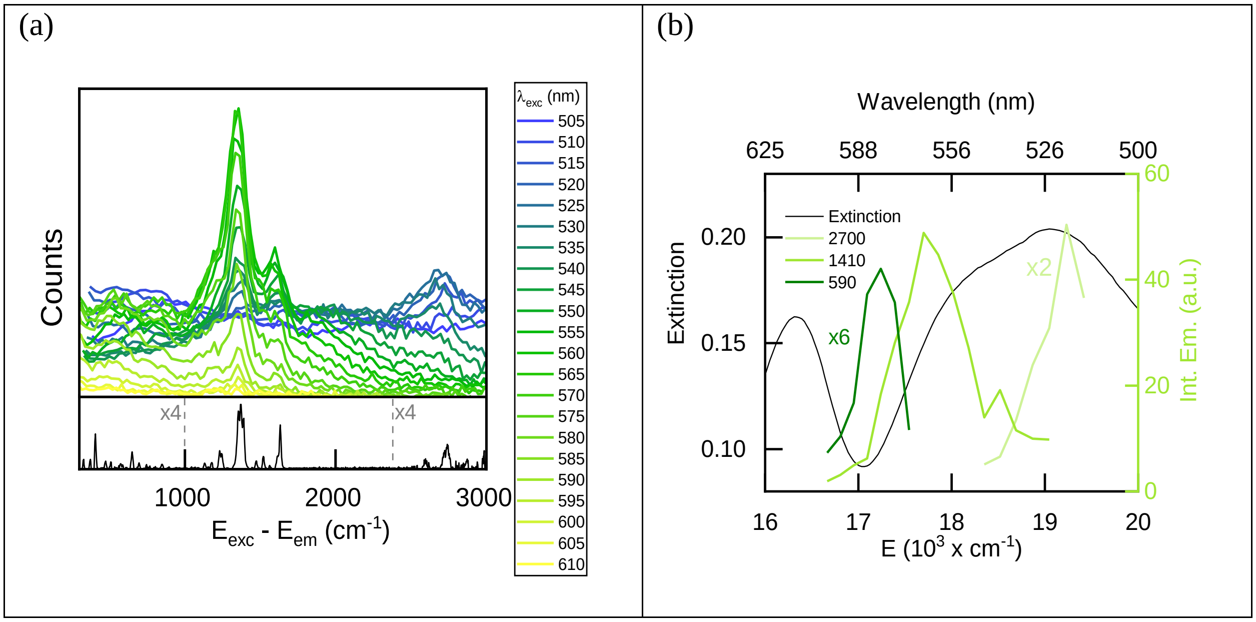

Figure S18: a) Raw emission spectra (background + VAS/SERS) in function of E_{exc}-E_{em} of CPM 4. The Raman spectrum of PIC is plotted below, with the regions below 1000 cm^{-1} and above 2500 cm^{-1} scaled by a factor x4. The emission is plotted from λ_{exc} = 505 nm (blue line) to 610 nm (yellow line) every 5 nm. b) Integrated emission of the signals from the region centered at 590 cm^{-1} (dark green curve, scaled by a factor of 6), 1410 cm^{-1} (green curve), and 2700 cm^{-1} (light green curve, scaled by a factor of 2) in function of the excitation wavelength. The extinction spectrum of CPM 4 is superposed and the scalebar reported on the left.

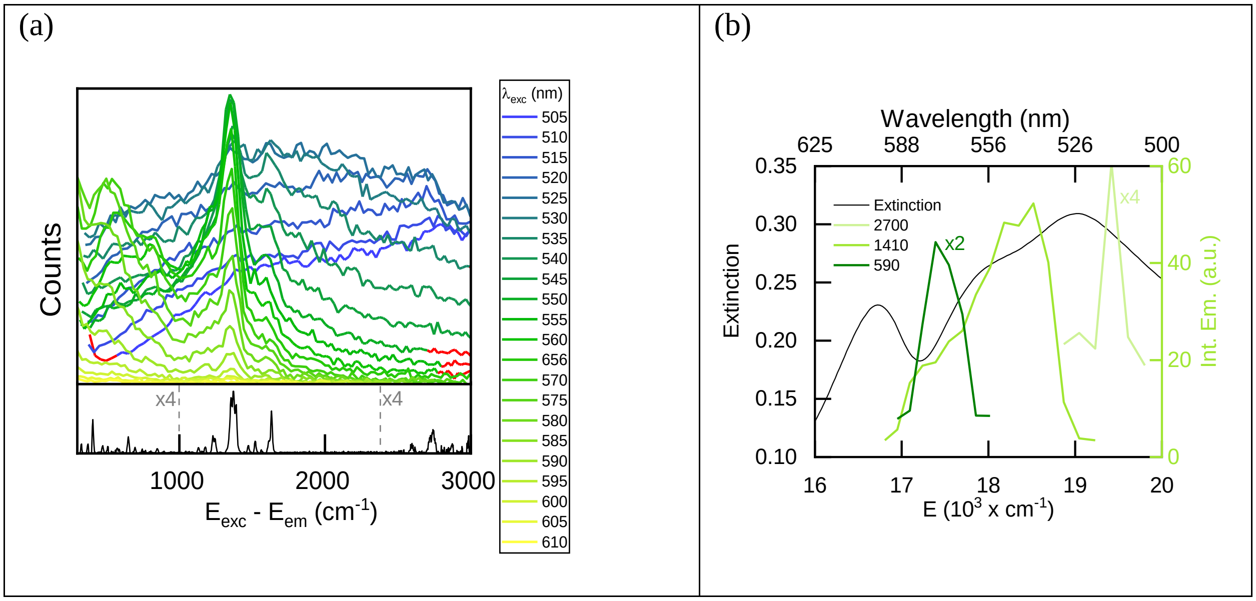

Figure S19: a) Raw emission spectra (background + VAS/SERS) in function of E_{exc}-E_{em} of CPM 5. The Raman spectrum of PIC is plotted below, with the regions below 1000 cm^{-1} and above 2500 cm^{-1} scaled by a factor x4. The emission is plotted from λ_{exc} = 505 nm (blue line) to 610 nm (yellow line) every 5 nm. b) Integrated emission of the signals from the region centered at 590 cm^{-1} (dark green curve, scaled by a factor of 2), 1410 cm^{-1} (green curve), and 2700 cm^{-1} (light green curve, scaled by a factor of 4) in function of the excitation wavelength. The extinction spectrum of CPM 5 is superposed and the scalebar reported on the left.

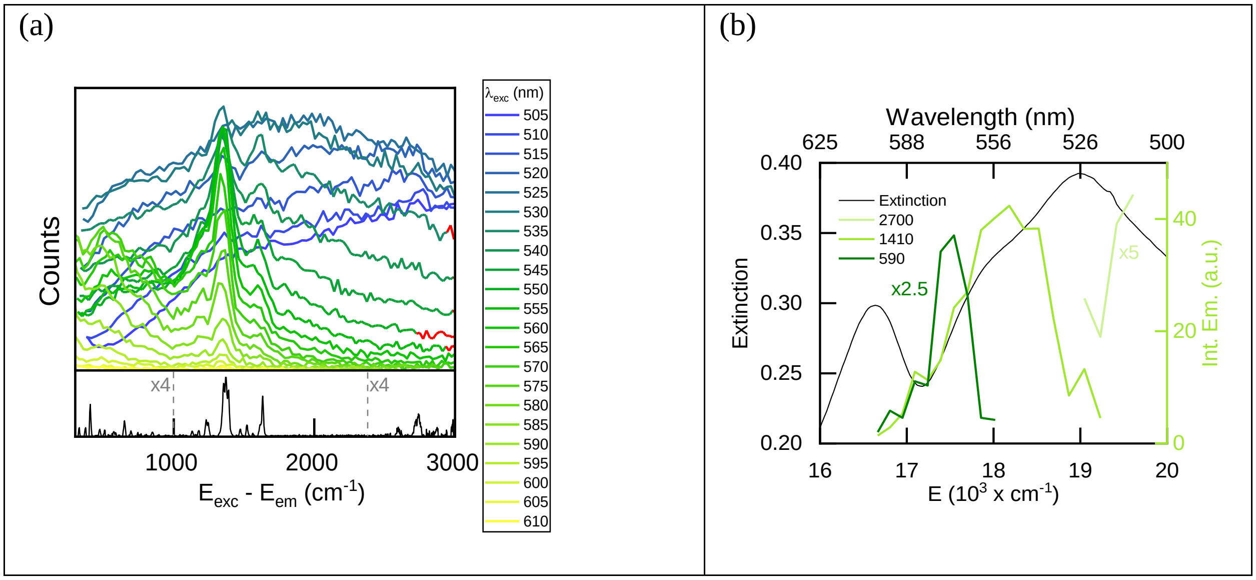

Figure S20: a) Raw emission spectra (background + VAS/SERS) in function of E_{exc}-E_{em} of CPM 6. The Raman spectrum of PIC is plotted below, with the regions below 1000 cm^{-1} and above 2500 cm^{-1} scaled by a factor x4. The emission is plotted from λ_{exc} = 505 nm (blue line) to 610 nm (yellow line) every 5 nm. b) Integrated emission of the signals from the region centered at 590 cm^{-1} (dark green curve, scaled by a factor of 2.5), 1410 cm^{-1} (green curve), and 2700 cm^{-1} (light green curve, scaled by a factor of 5) in function of the excitation wavelength. The extinction spectrum of CPM 6 is superposed and the scalebar reported on the left.

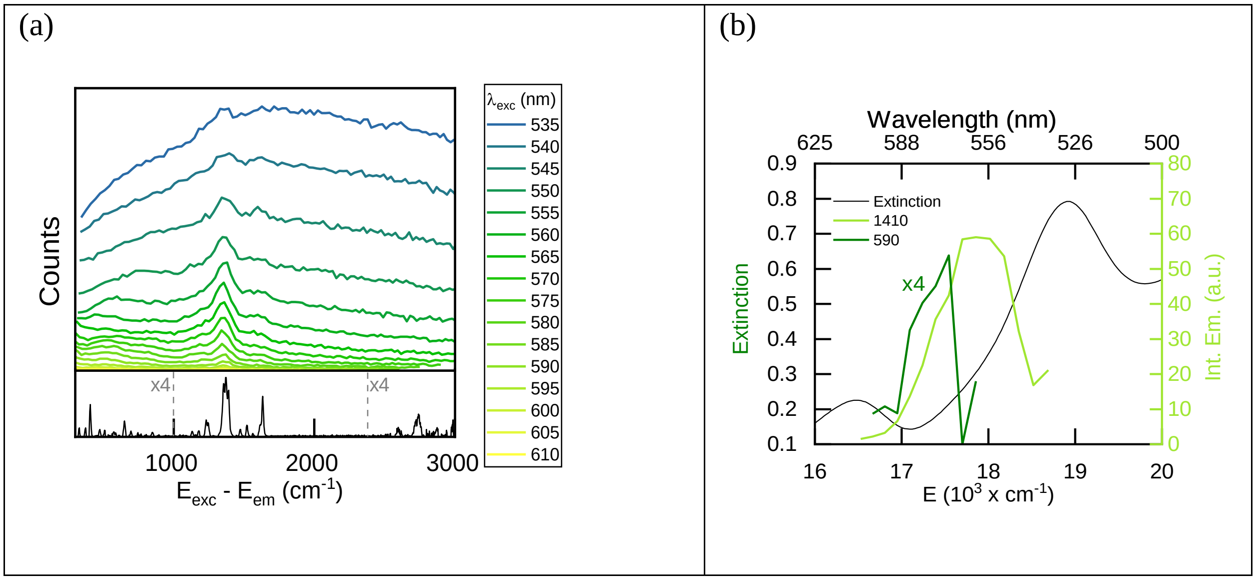

Figure S21: a) Raw emission spectra (background + VAS/SERS) in function of E_{exc}-E_{em} of CPM 7. The emission is measured exciting from 535 nm (blue line) to 610 nm (yellow line) every 5 nm. The emission is plotted from λ_{exc} = 535 nm (blue line) to 610 nm (yellow line) every 5 nm. The Raman spectrum of PIC is plotted below, with the regions below 1000 cm^{-1} and above 2500 cm^{-1} scaled by a factor x4. b) Integrated emission of the signals from the region centered at 590 cm^{-1} (dark green curve, scaled by a factor of 4), and 1410 cm^{-1} (green curve), in function of the excitation wavelength. The extinction spectrum of CPM 7 is superposed and the scalebar reported on the left.

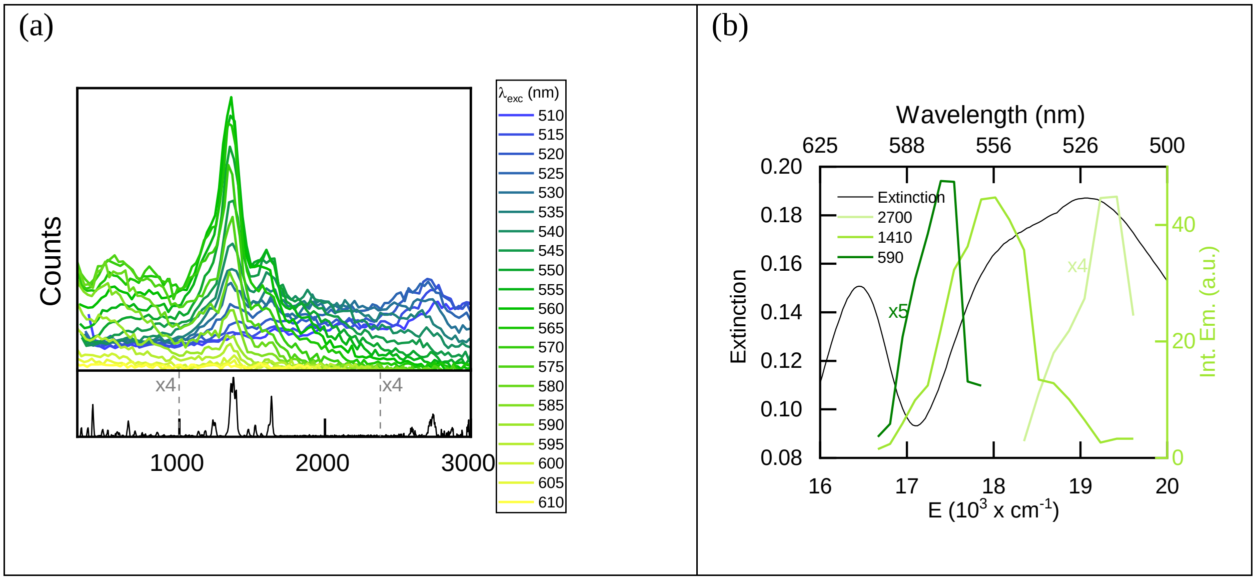

Figure S22: a) Raw emission spectra (background + VAS/SERS) in function of E_{exc}-E_{em} of CPM 8. The Raman spectrum of PIC is plotted below, with the regions below 1000 cm^{-1} and above 2500 cm^{-1} scaled by a factor x4. The emission is plotted from λ_{exc} = 505 nm (blue line) to 610 nm (yellow line) every 5 nm. b) Integrated emission of the signals from the region centered at 590 cm^{-1} (dark green curve, scaled by a factor of 5), 1410 cm^{-1} (green curve), and 2700 cm^{-1} (light green curve, scaled by a factor of 4) in function of the excitation wavelength. The extinction spectrum of CPM 8 is superposed and the scalebar reported on the left.

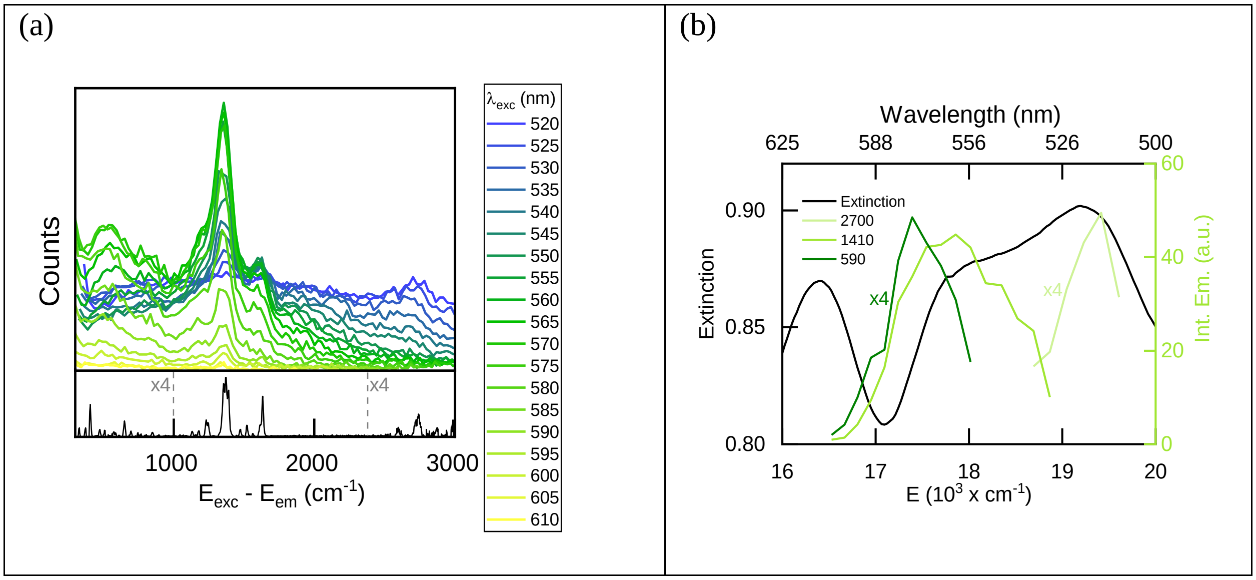

Figure S23: a) Raw emission spectra (background + VAS/SERS) in function of E_{exc}-E_{em} of CPM 9. The Raman spectrum of PIC is plotted below, with the regions below 1000 cm^{-1} and above 2500 cm^{-1} scaled by a factor x4. The emission is plotted from λ_{exc} = 505 nm (blue line) to 610 nm (yellow line) every 5 nm. b) Integrated emission of the signals from the region centered at 590 cm^{-1} (dark green curve, scaled by a factor of 4), 1410 cm^{-1} (green curve), and 2700 cm^{-1} (light green curve, scaled by a factor of 4) in function of the excitation wavelength. The extinction spectrum of CPM 9 is superposed and the scalebar reported on the left.

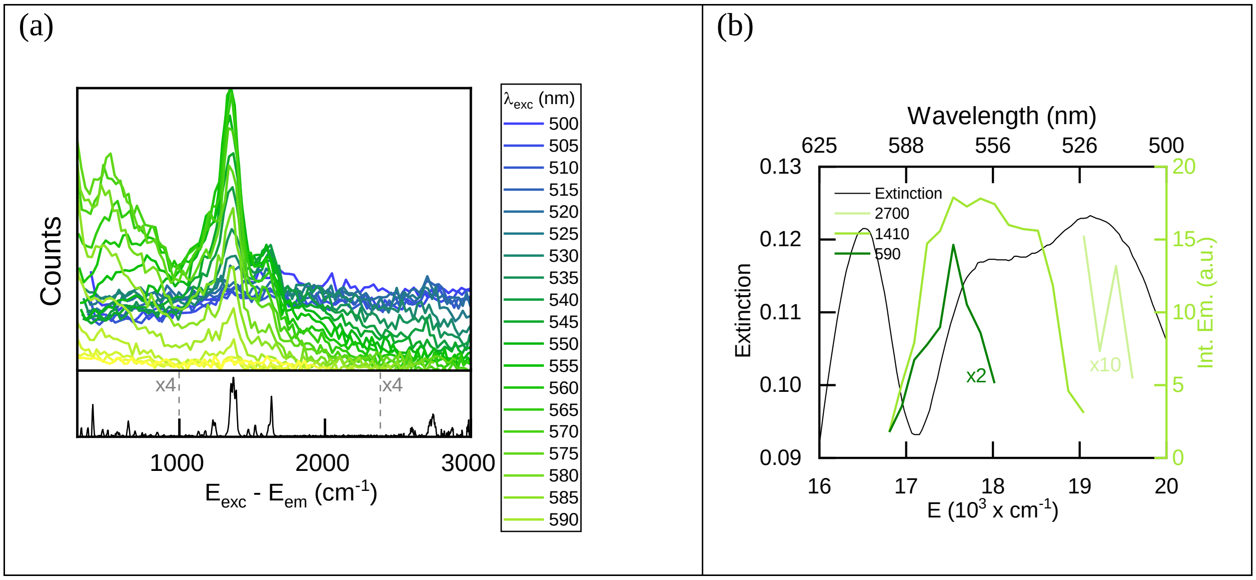

Figure S24: a) Raw emission spectra (background + VAS/SERS) in function of E_{exc}-E_{em} of CPM 10. The Raman spectrum of PIC is plotted below, with the regions below 1000 cm^{-1} and above 2500 cm^{-1} scaled by a factor x4. The emission is plotted from λ_{exc} = 505 nm (blue line) to 610 nm (yellow line) every 5 nm. b) Integrated emission of the signals from the region centered at 590 cm^{-1} (dark green curve, scaled by a factor x2), 1410 cm^{-1} (green curve), and 2700 cm^{-1} (light green curve, scaled by a factor of 10) in function of the excitation wavelength. The extinction spectrum of CPM 10 is superposed and the scalebar reported on the left.

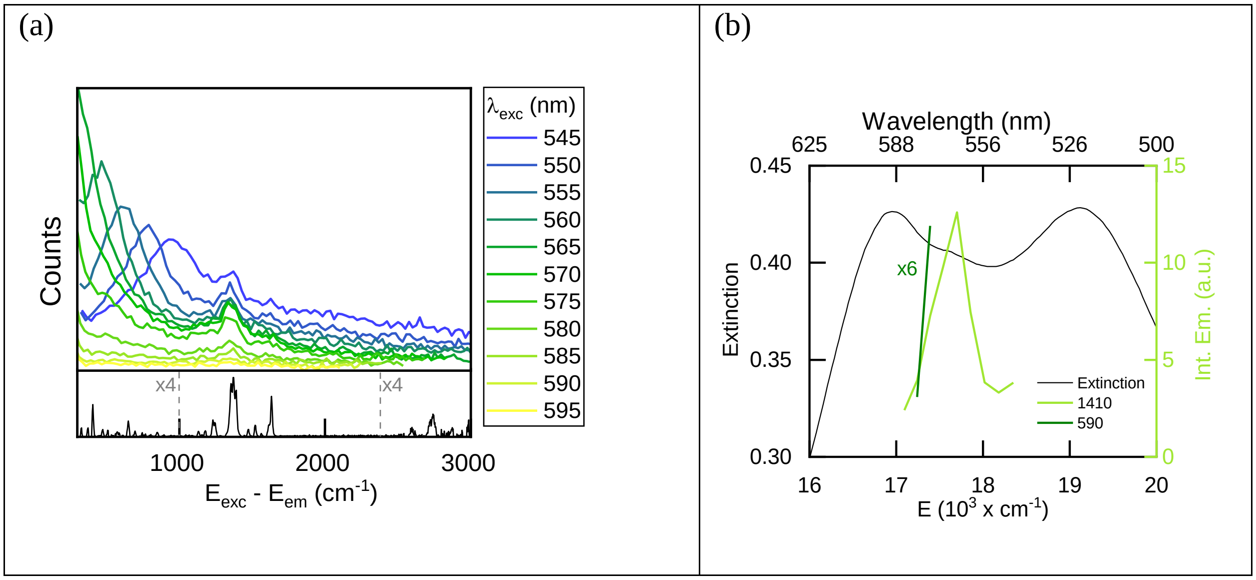

Figure S25: a) Raw emission spectra (background + VAS/SERS) in function of E_{exc}-E_{em} of CPM 11. The emission is plotted from λ_{exc} = 545 nm (blue line) to 610 nm (yellow line) every 5 nm. The Raman spectrum of PIC is plotted below, with the regions below 1000 cm^{-1} and above 2500 cm^{-1} scaled by a factor x4. b) Integrated emission of the signals from the region centered at 590 cm^{-1} (dark green curve, scaled by a factor of 6), and 1410 cm^{-1} (green curve) in function of the excitation wavelength. The extinction spectrum of CPM 11 is superposed and the scalebar reported on the left. Only two data points were obtained from the weak signal from the region centered at 590 cm^{-1}.

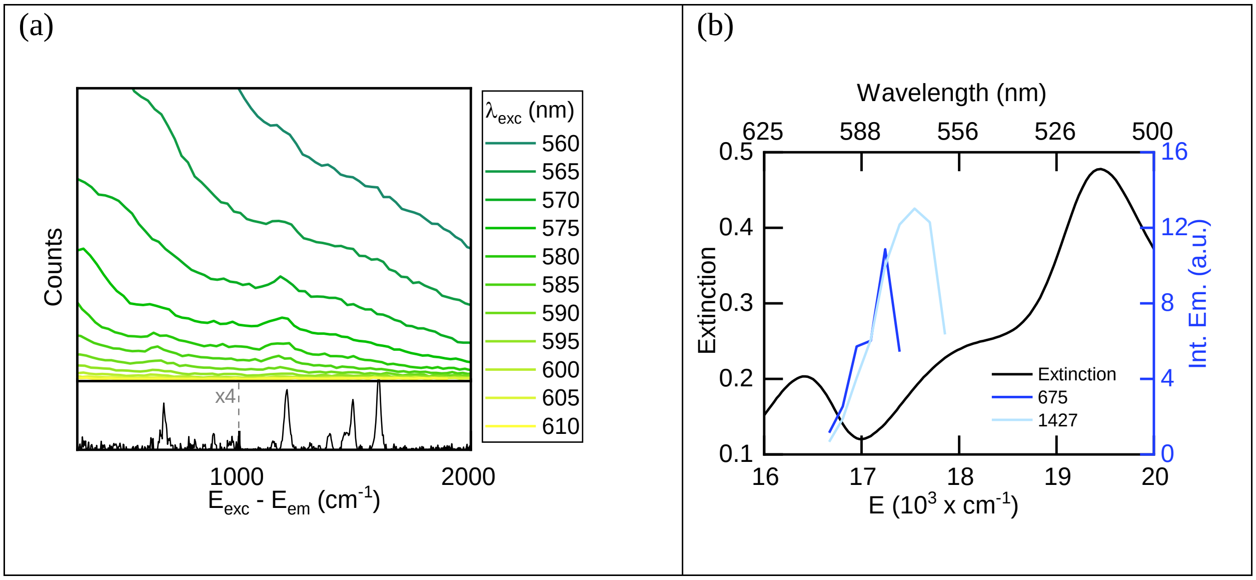

Figure S26: a) Raw emission spectra (background + VAS/SERS) in function of E_{exc}-E_{em} of CPM 13. The emission is plotted from λ_{exc} = 560 nm (green line) to 610 nm (yellow line) every 5 nm. The Raman spectrum of TDBC is plotted below, with the region below 1000 cm^{-1} scaled by a factor x4. b) Integrated emission of the signals from the region centered at 675 cm^{-1} (dark blue curve), and 1427 cm^{-1} (light blue curve) in function of the excitation wavelength. The extinction spectrum of CPM 13 is superposed and the scalebar reported on the left.

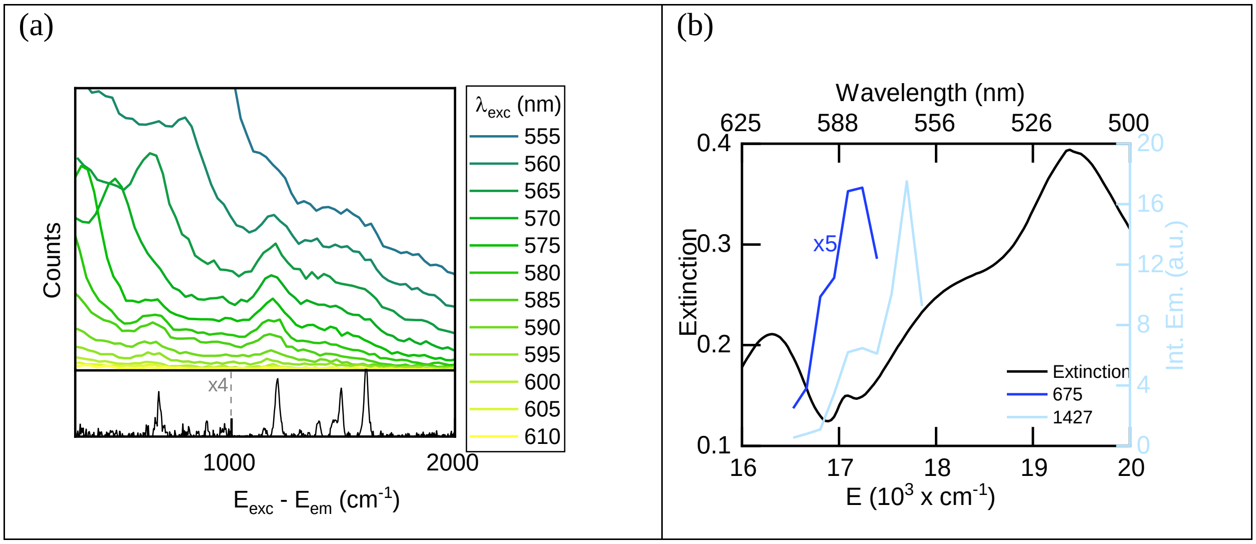

Figure S27: a) Raw emission spectra (background + VAS/SERS) in function of E_{exc}-E_{em} of CPM 14. The emission is plotted from λ_{exc} = 555 nm (blue line) to 610 nm (yellow line) every 5 nm. The Raman spectrum of TDBC is plotted below, with the region below 1000 cm^{-1} scaled by a factor x4. b) integrated emission of the signals from the region centered at 675 cm^{-1} (dark blue curve, scaled by a factor of 5), and 1427 cm^{-1} (light blue curve) in function of the excitation wavelength. The integration values are scaled by a factor x1000. The extinction spectrum of CPM 14 is superposed and the scalebar reported on the left.

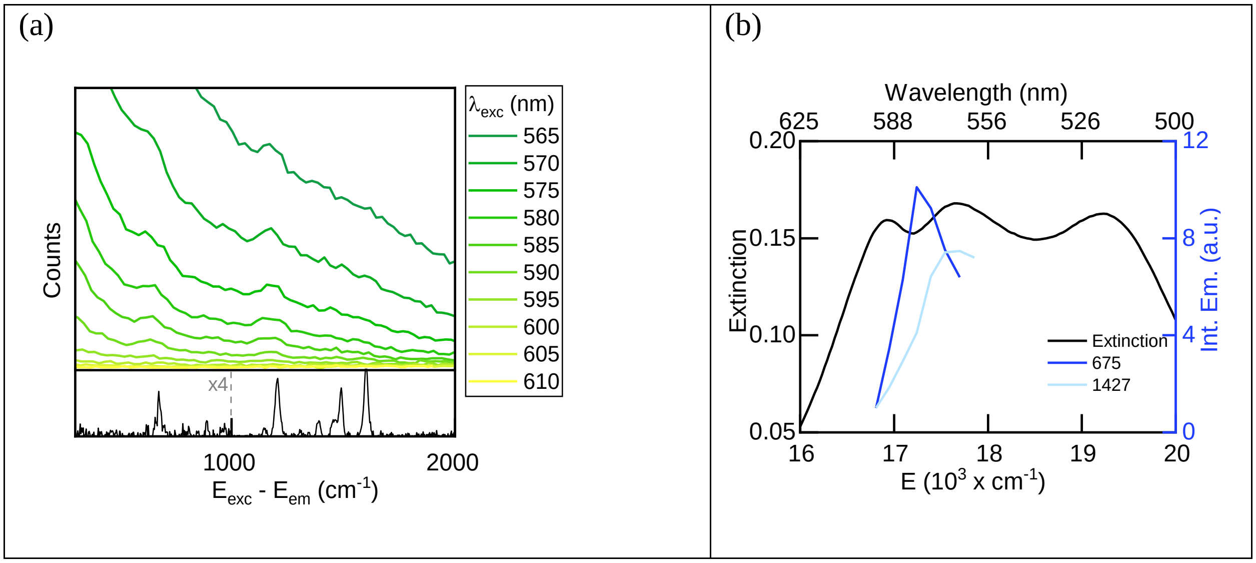

Figure S28: a) Raw emission spectra (background + VAS/SERS) in function of E_{exc}-E_{em} of CPM 15. The emission is plotted from λ_{exc} = 565 nm (green line) to 610 nm (yellow line) every 5 nm. The Raman spectrum of TDBC is plotted below, with the region below 1000 cm^{-1} scaled by a factor x4. b) integrated emission of the signals from the region centered at 675 cm^{-1} (dark blue curve), and 1427 cm^{-1} (light blue curve) in function of the excitation wavelength. The extinction spectrum of CPM 15 is superposed and the scalebar reported on the left.

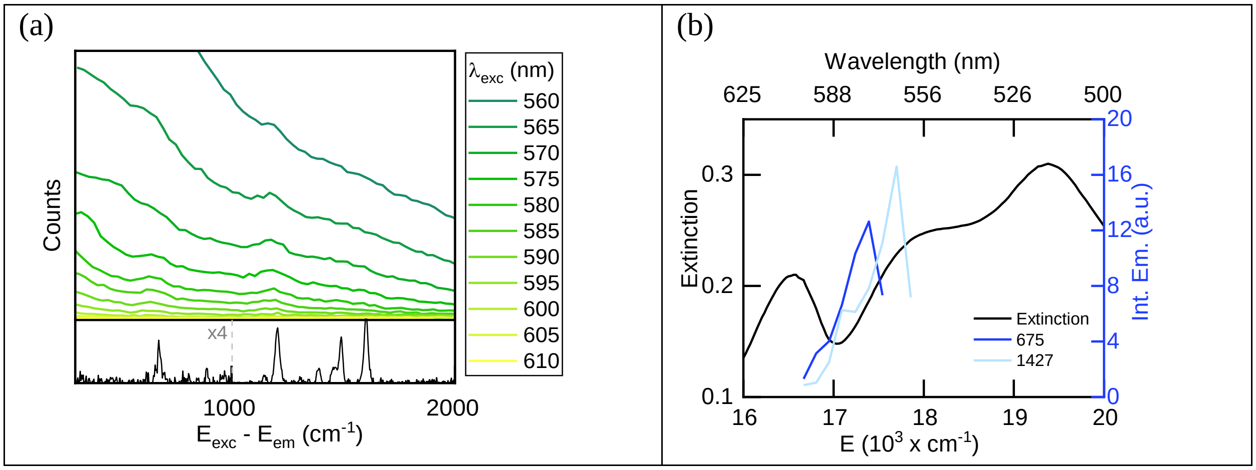

Figure S29: a) Raw emission spectra (background + VAS/SERS) in function of E_{exc}-E_{em} of CPM 16. The emission is plotted from λ_{exc} = 570 nm (green line) to 610 nm (yellow line) every 5 nm. The Raman spectrum of TDBC is plotted below, with the region below 1000 cm^{-1} scaled by a factor x4. b) Integrated emission of the signals from the region centered at 675 cm^{-1} (dark blue curve), and 1427 cm^{-1} (light blue curve) in function of the excitation wavelength. The extinction spectrum of CPM 16 is superposed and the scalebar reported on the left.

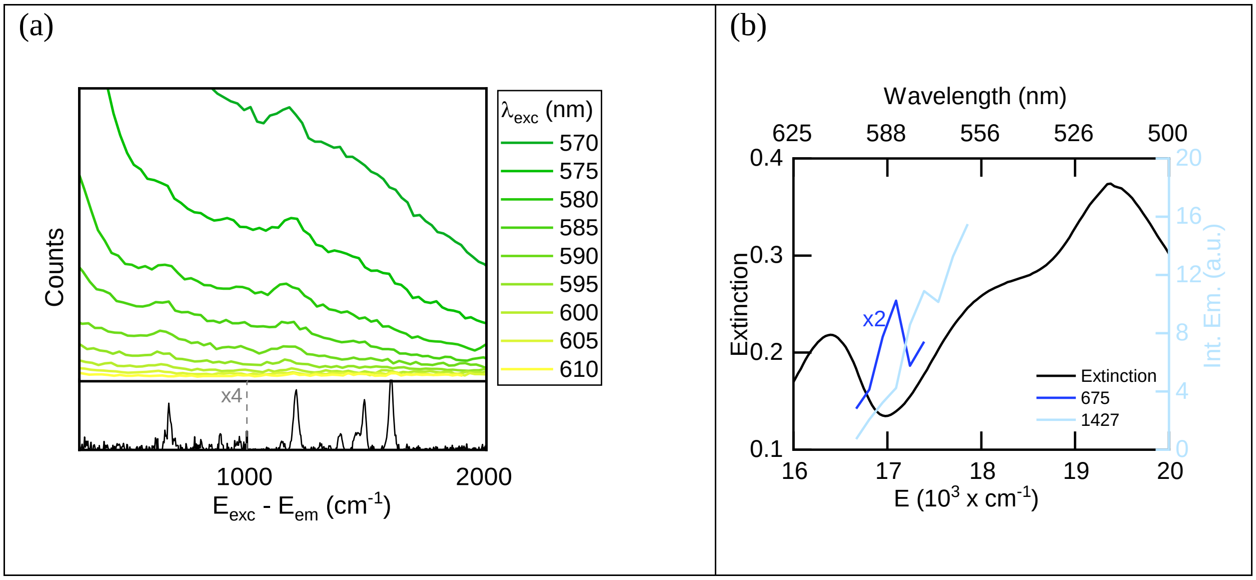

Figure S30: a) Raw emission spectra (background + VAS/SERS) in function of E_{exc}-E_{em} of CPM 17. The emission is plotted from λ_{exc} = 570 nm (green line) to 610 nm (yellow line) every 5 nm. The emission is plotted from λ_{exc} = 570 nm (green line) to 610 nm (yellow line) every 5 nm. The Raman spectrum of TDBC is plotted below, with the region below 1000 cm^{-1} scaled by a factor x4. b) Integrated emission of the signals from the region centered at 675 cm^{-1} (dark blue curve, scaled by a factor of 2), and 1427 cm^{-1} (light blue curve) in function of the excitation wavelength. The extinction spectrum of CPM 17 is superposed and the scalebar reported on the left.

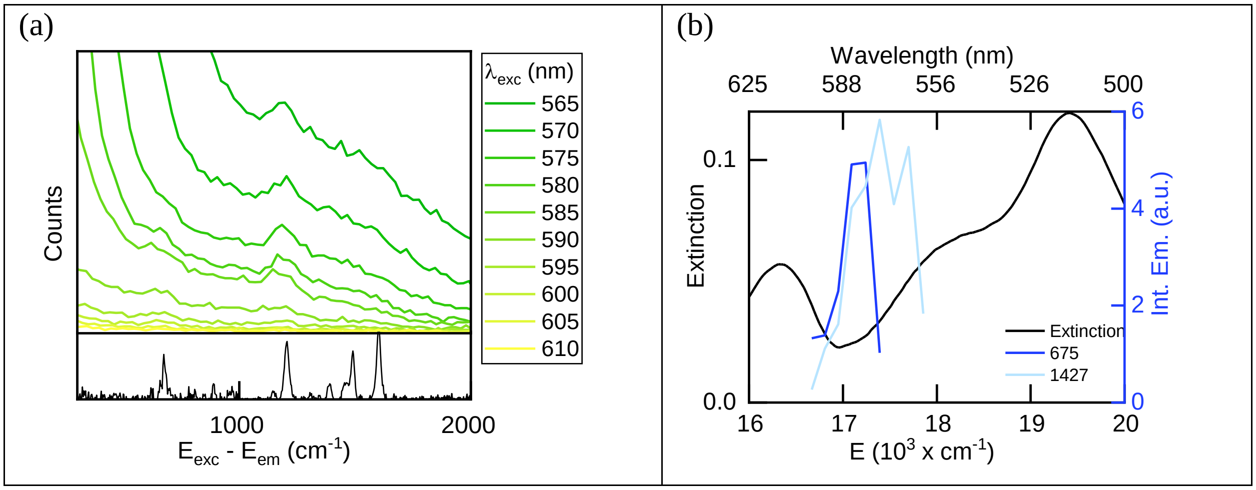

Figure S31: a) Raw emission spectra (background + VAS/SERS) in function of E_{exc}-E_{em} of CPM 18. The emission is plotted from λ_{exc} = 565 nm (green line) to 610 nm (yellow line) every 5 nm.The Raman spectrum of TDBC is plotted below, with the region below 1000 cm^{-1} scaled by a factor x4. b) Integrated emission of the signals from the region centered at 675 cm^{-1} (dark blue curve), and 1427 cm^{-1} (light blue curve) in function of the excitation wavelength. The extinction spectrum of CPM 14 is superposed and the scalebar reported on the left.

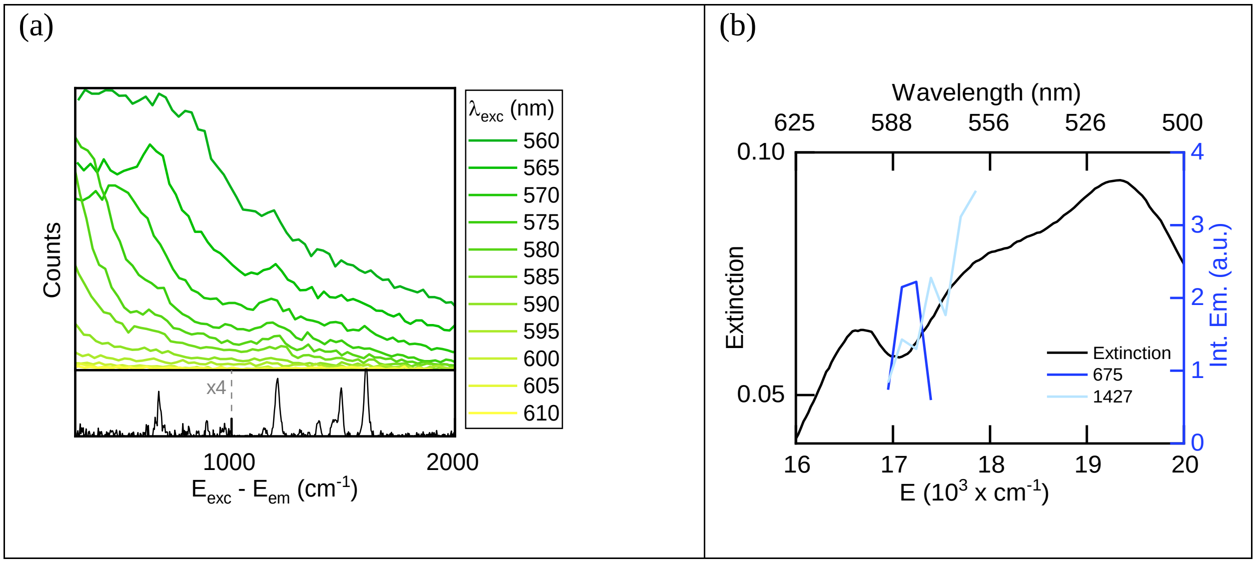

Figure S32: a) Raw emission spectra (background + VAS/SERS) in function of E_{exc}-E_{em} of CPM 19. The emission is plotted from λ_{exc} = 560 nm (green line) to 610 nm (yellow line) every 5 nm. The Raman spectrum of TDBC is plotted below, with the region below 1000 cm^{-1} scaled by a factor x4. b) Integrated emission of the signals from the region centered at 675 cm^{-1} (dark blue curve), and 1427 cm^{-1} (light blue curve) in function of the excitation wavelength. The extinction spectrum of CPM 13 is superposed and the scalebar reported on the left.

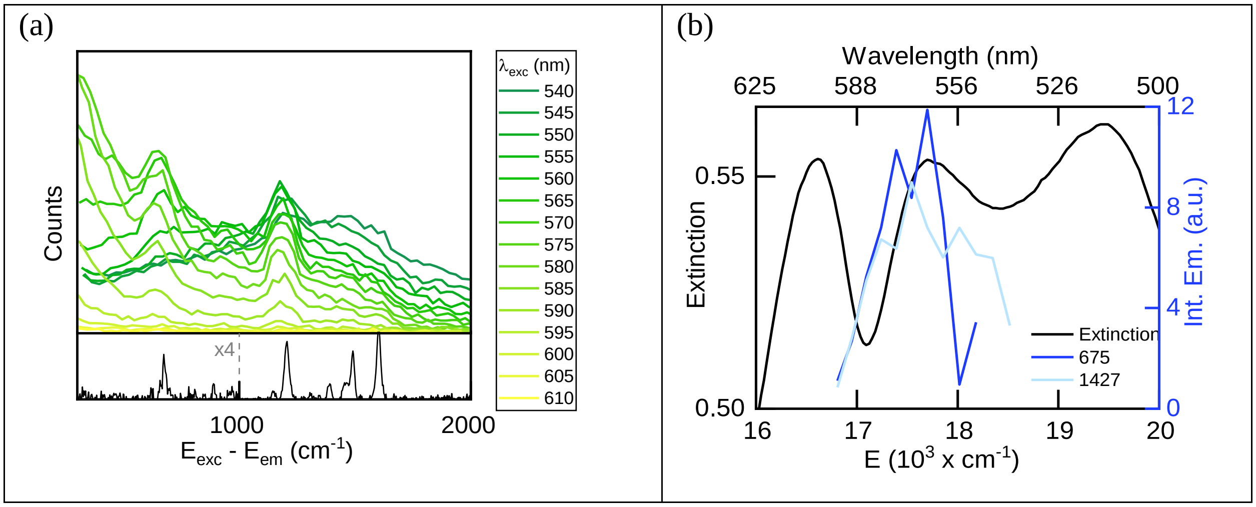

Figure S33: a) Raw emission spectra (background + VAS/SERS) in function of E_{exc}-E_{em} of CPM 20. The emission is plotted from λ_{exc} = 540 nm (green line) to 610 nm (yellow line) every 5 nm. The Raman spectrum of TDBC is plotted below, with the region below 1000 cm^{-1} scaled by a factor x4. b) Integrated emission of the signals from the region centered at 675 cm^{-1} (dark blue curve), and 1427 cm^{-1} (light blue curve) in function of the excitation wavelength. The extinction spectrum of CPM 20 is superposed and the scalebar reported on the left.

5. Control emission experiment with PIC/TDBC bound to long NR@PSS

As a control experiment, longer NR@PSS were bonded with PIC and TDBC and their emission were analyzed. Longer NR@PSS have both the transversal and longitudinal plasmons detuned from the J aggregate absorption, and thus, no strong coupling can be achieved. For this experiment, long NR reported in Figures S1 and S2 were loaded with 2.3 mg/mL of PSS, with the same procedure reported for CPMs. In order to have a number of PIC (or TDBC) on the surface of the NR@PSS in the same order of magnitude as for the CPMs reported in the sections above, two solutions with [long NR@PSS] = 20 nM and [PIC] = 0.2 μM (or [TDBC] = 3.1 μM) were prepared. The extinction spectra of the solutions did not show any plexcitons (Figure S34). The two samples were excited at the same wavelengths as used for CPMs. In addition, the samples were excited in the transversal plasmon region, between 775 nm and 810 nm (Figure S35), and the emission was recorded up to 880 nm. No VAS nor SERS patterns were observed.

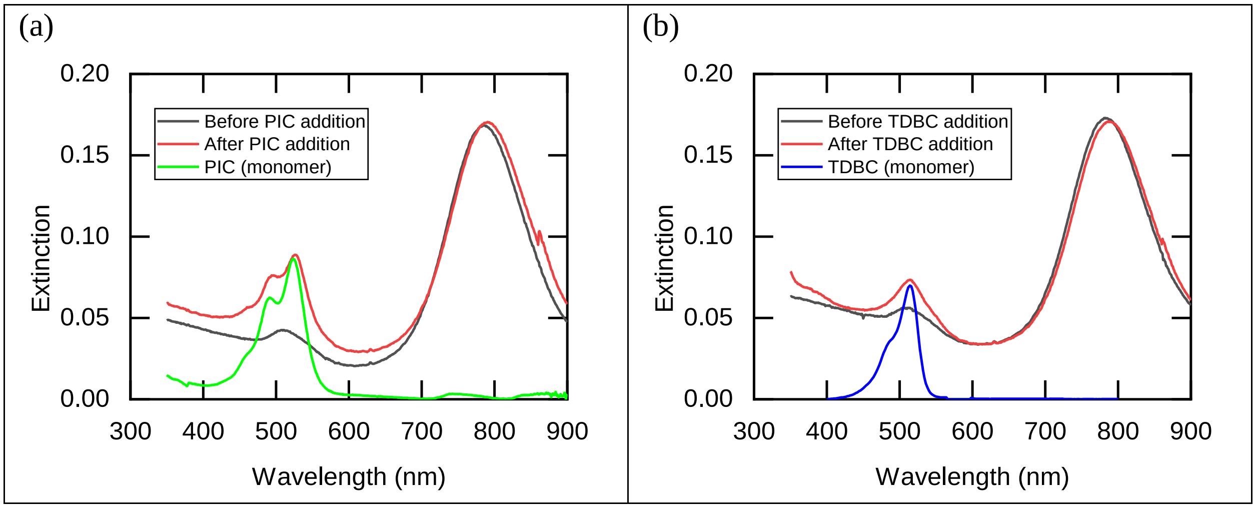

Figure S34: a) Long NR@PSS before and after the addition of PIC, a spectrum of the monomeric PIC is included and normalized at the extinction value of the PIC-NRs hybrid at 525 nm; b) Long NR@PSS before and after the addition of TDBC, a spectrum of the monomeric TDBC is included and normalized at the extinction value of the TDBC-NRs hybrid at 515 nm.

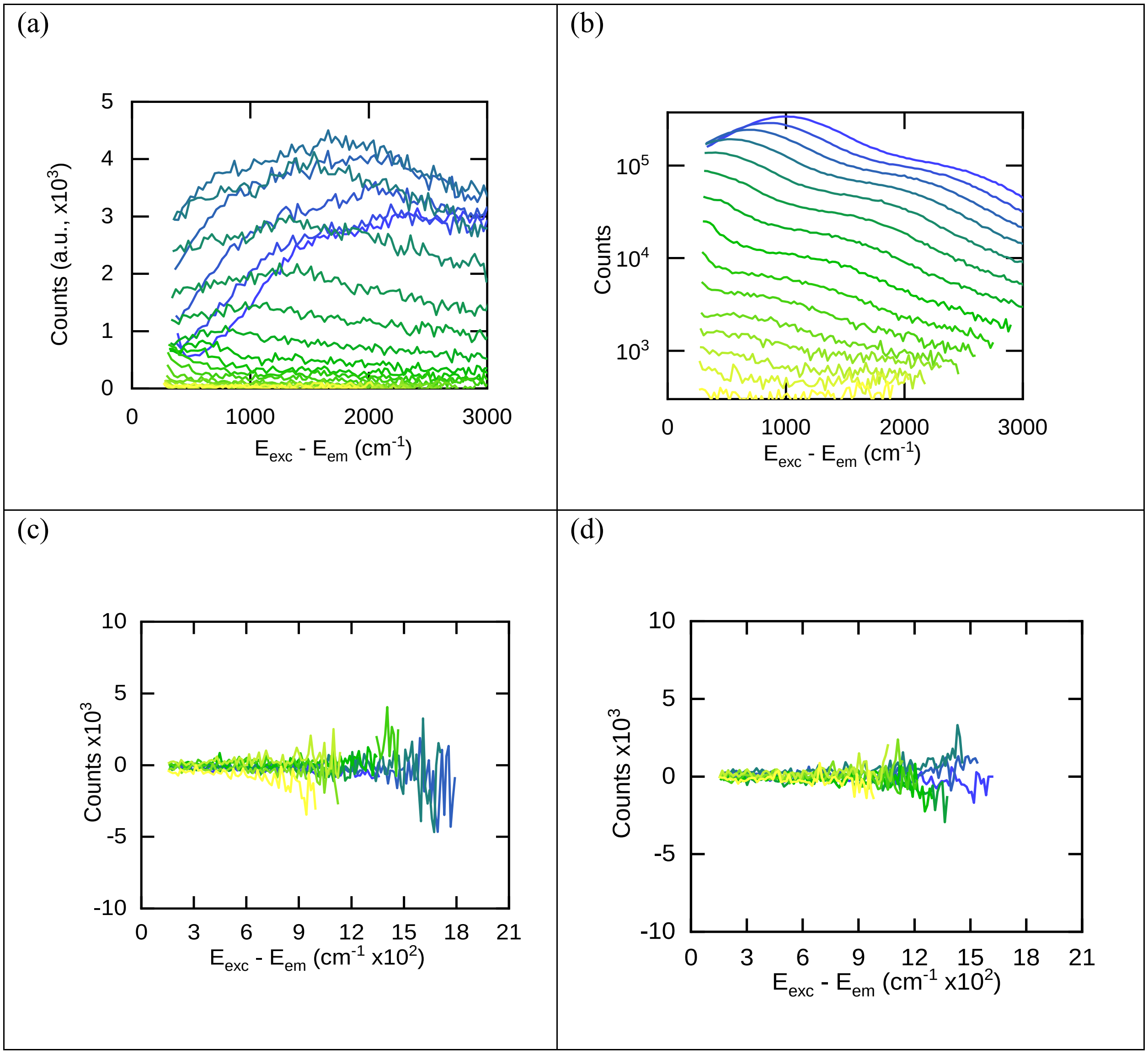

Figure S35: a) Emission spectra as a function of E_{exc}-E_{em} of long NR@PSS-PIC excited from 505 nm (blue line) to 610 nm (yellow line) every 5 nm; b) same as a), but for long NR@PSS-PIC excited from 540 nm (blue line) to 610 nm (yellow line). The emission is plotted in logarithmic scale because of the high background emission; c) Emission spectra as a function of E_{exc}-E_{em} of long NR@PSS-PIC excited from 770 nm (blue line) to 810 nm (yellow line); d) same as c), but for long NR@PSS-TDBC.

6. Further information about the calculation of the W_{VAS}

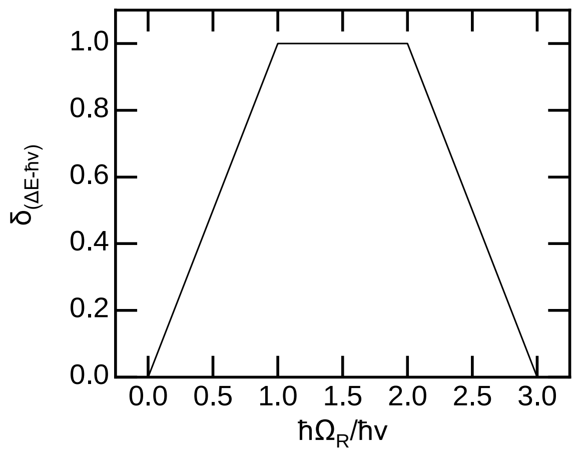

Figure S36: Dependence of the $\delta_{(\Delta E\text{-}\hbar\nu)}$ on the $\hbar\Omega_R/(\hbar\nu)$ ratio.

Table S4: The CPMs for which W_{VAS} was calculated and the respective values of the oscillator strength f, number of coupled excitons N, and Rabi splitting $\hbar\Omega_R$.

**CPM**	**f**	**N**	**$\hbar\Omega_R$ (cm^{-1})**
**1**	0.0475	16	1210
**2**	0.055	18	1269
**3**	0.095	32	1475
**4**	0.1	33	1512
**5**	0.03	10	821
**6**	0.035	12	1029
**7**	0.0825	28	1383
**8**	0.09	30	1310
**12**	0.02	20	671
**13**	0.075	75	1310
**14**	0.1	100	1608
**15**	0.006	6	589
**16**	0.05	50	1018
**17**	0.08	80	1337
**18**	0.09	90	1301
**19**	0.035	35	944

7. Further information on the comparison between emission, EF_{SERS}, W_{VAS}, and the emission

Among the 20 CPMs presented, the emission of CPMs 1-8, and 12 – 19 were compared with the calculated values of W_{VAS} and EF_{SERS}. The values of their non-normalized emission, EF_{SERS} and W_{VAS} are reported in Table S5-8. The non-normalized values of EF_{SERS} and W_{VAS} were also plotted in Figure S37 and Figure S38 to ease the understanding of their trends. Being the process of relaxation from the DS to the LP active only for $\hbar\Omega_R/(\hbar\nu) > 1.5$, W_{VAS} was not calculated when $\hbar\Omega_R/(\hbar\nu) < 1.5$.

Table S5: Values of $\hbar\Omega_R/(\hbar\nu)$, maximum of integrated emission, W_{VAS}, EF_{SERS} for CPMs prepared with PIC and for the vibrational region centered at 590 cm^{-1}.

$\hbar\Omega_R/(\hbar\nu)$	CPM	Max of int. em.	Max of int. em. errorbar	W_{VAS} (10^9 * s^{-1})	EF_{SERS}
**1.39**	5	22884	249	679	7180
**1.74**	6	14831	256	582	2997
**2.05**	1	16085	190	429	1987
**2.15**	2	13927	647	314	1412
**2.22**	8	11442	287	177	413
**2.34**	7	13449	473	162	479
**2.5**	3	8972	344	107	604
**2.56**	4	7425	179	89	483

Table S6: Values of $\hbar\Omega_R/(\hbar\nu)$, maximum of integrated emission, W_{VAS}, EF_{SERS} for CPMs prepared with PIC and for the vibrational region centered at 1410 cm^{-1}.

**$\hbar\Omega_R/(\hbar\nu)$**	**CPM**	**Max of int. em.**	**Max of int. em. errorbar**	**W_{VAS} (10^9 * s^{-1})**	**EF_{SERS}**
**0.58**	5	41362	685	107	2263
**0.73**	6	37937	683	99	5048
**0.86**	1	54325	483	64	10810
**0.9**	2	46355	700	49	9730
**0.93**	8	42138	622	19	13647
**0.98**	7	55973	1042	24	15800
**1.05**	3	72392	1343	18	13156
**1.07**	4	47968	444	16	11681

Table S7: Values of $\hbar\Omega_R/(\hbar\nu)$, maximum of integrated emission, W_{VAS}, EF_{SERS} for CPMs prepared with TDBC and for the vibrational region centered at 675 cm^{-1}.

**$\hbar\Omega_R/(\hbar\nu)$**	**CPM**	**Max of int. em.**	**Max of int. em. errorbar**	**W_{VAS} (10^9 * s^{-1})**	**EF_{SERS}**
**0.9**	15	10107	458		35862
**1.0**	12	14463	672		24030
**1.5**	16	12641	683	174	6284
**1.9**	13	10871	750	96.7	3539
**1.93**	18	8371	275	116	2210
**2.0**	17	5121	441	109	1683
**2.4**	14	3419	183	53.8	1160

Table S8: Values of $\hbar\Omega_R/(\hbar\nu)$, maximum of integrated emission, W_{VAS}, EF_{SERS} for CPMs prepared with TDBC and for the vibrational region centered at 1427 cm^{-1}.

**$\hbar\Omega_R/(\hbar\nu)$**	**CPM**	**Max of int. em.**	**Max of int. em. errorbar**	**W_{VAS} (10^9 * s^{-1})**	**EF_{SERS}**
**0.4**	15	7423	737	214	18199
**0.5**	12	11279	1112	22	22052
**0.66**	19	11236	298	11	25368
**0.7**	16	16304	1764	5.3	26561
**0.91**	18	13025	442	2.1	27747
**0.92**	13	13015	617	3.05	35442
**0.94**	17	15391	1639	2.74	14629
**1.13**	14	17200	246	1.87	30178

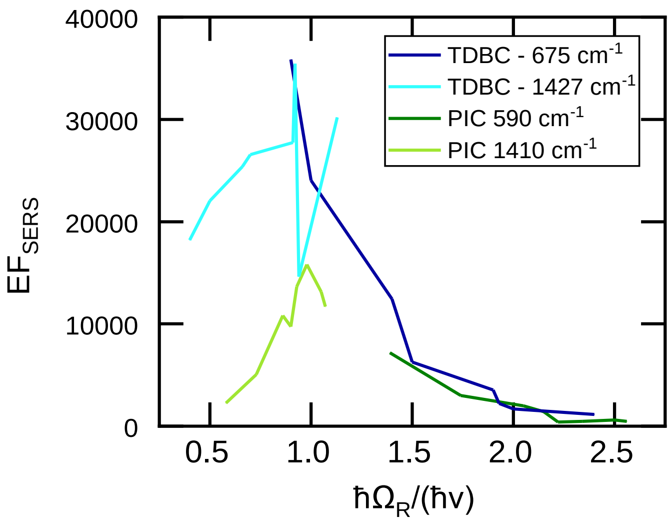

Figure S37: Calculated EF_{SERS} in function of $\hbar\Omega_R/(\hbar\nu)$.

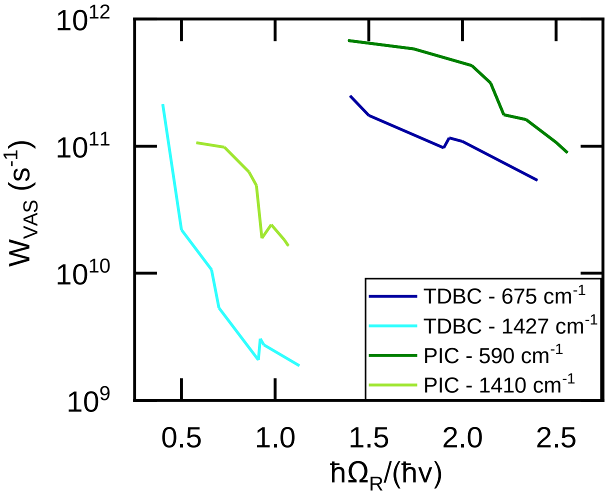

Figure S38: Calculated W_{VAS} in function of $\hbar\Omega_R/(\hbar\nu)$.

8. Preparation and characterization of the emission of Fabry-Pérot cavities coupled with TDBC J-aggregates (TDBC-FP)

Cavity preparation. The Fabry-Pérot cavity coupled with TDBC (TDBC-FP-1) consists of a sandwich structure where two Ag mirrors embed the TDBC layer. This structure is supported by a glass substrate (Figure S39a). The glass substrate (2.5 cm×2.5 cm) was cleaned via sonication for 15 min, in 1 % alkaline solution (Hellmanex in distilled water), followed by sonication for 1 hr in water and then ethanol. The cleaned glass substrate was dried in an oven overnight before the cavity fabrication. A thick Ag mirror (100 nm) was fabricated on top of the glass substrate by vacuum sputtering deposition (HEX, Korvus Technologies). Successively, the TDBC layer was spin-coated (WS-650-23B Spin Coater, Laurell) using an aqueous solution consisting of TDBC and poly(vinyl alcohol) (PVA). The cavity was finally closed via sputtering deposition of a semi-transparent Ag layer (30 nm).

The TDBC-PVA solution was prepared by adding PVA (5% in mass) to a 4 mM TDBC aqueous solution (Millipore water). The mixture was stirred at 80 °C for 2 hours to dissolve the PVA completely and filtered using a 0.2 μm pore-size filter. The filtered mixture was diluted 1.7 times with Millipore water and 300 μL were spin-coated on top of the 100 nm Ag layer at 1500 rpm.

Reflectivity measurements. The TDBC-FP-1 angle-dependent reflectivity was recorded on a Perkin Elmer LAMBDA 950 spectrometer equipped with a Universal Reflectance Accessory (Perkin Elmer). The angle θ was scanned from 10° to 50° with a step of 5°, and the wavelength was measured from 400 nm to 800 nm with a step of 1 nm. The reflectivity of TDBC-FP-1 is shown in Figure S39b.

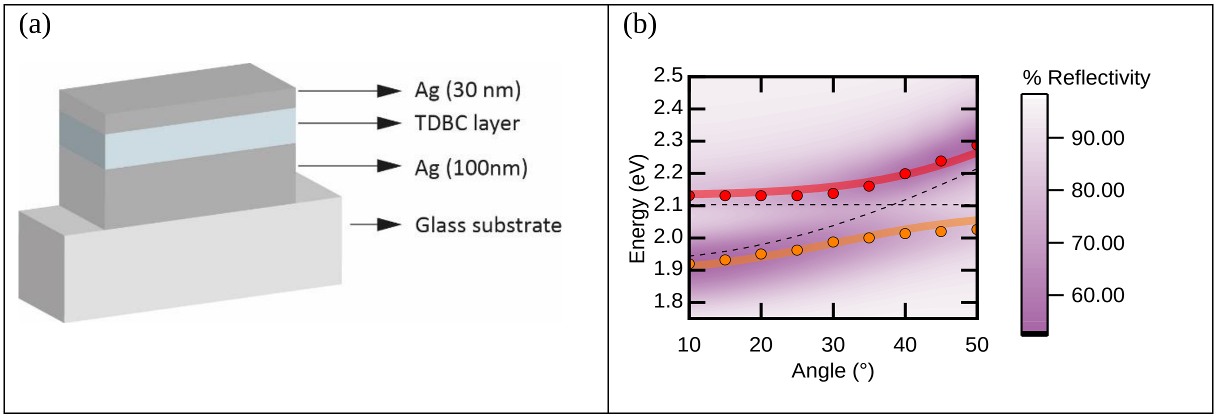

Figure S39: a) Structure of the TDBC-FP-1. b) Angle-dependent reflectivity of TDBC-FP-1. The UP and LP maxima are reported as red and orange circles, respectively. The UP and LP branches obtained with the coupled harmonic oscillator model are reported as red and orange lines, respectively. E_{exc} is reported as a horizontal black dashed line, and E_{cav} as a black dashed line.

The angle-dependent reflectivity was fitted using a coupled harmonic oscillator model:

$$E_{UP/LP}\left(k_{||}\right) = \frac{1}{2}\left(E_{exc} + E_{cav}(k_{||})\right) \pm \sqrt{\left(\frac{\hbar\Omega_R}{2}\right)^2 + \frac{1}{4}\left(E_{exc} - E_{cav}\left(k_{||}\right)\right)^2} \qquad \text{S1}$$

where E_{UP} and E_{LP} are the energies of the UP and LP, respectively, E_{exc} is the exciton energy, E_{cav} is the energy of the cavity, which is dependent on the angle θ according to the following equations:

$$E_{cav}\left(k_{||}\right) = \frac{\hbar c}{n_{eff}}\sqrt{k_{||}^2 + \left(\frac{\pi}{2 * L_{cav}}\right)^2} \qquad \text{S2}$$

$$k_{||} = \frac{2\pi}{\lambda}\sin(\theta) \qquad \text{S3}$$

with L_{cav} being the cavity thicknesses, and n_{eff} the refractive index. The results of the fitting were: $\hbar\Omega_R$ = 155 meV, L_{cav} = 205 nm, n_{eff} = 1.563, and E_{exc} = 2.103 eV (589 nm). The E_{exc} value retrieved from the fitting agrees with the absorption value (589 nm). The calculated UP, LP, and E_{exc} were added in Figure S39b (red, orange, and black dashed horizontal lines, respectively). E_{cav} was calculated using Eq. 2 and reported in Figure S39b (black dashed line).

Comparison between the emission of TDBC-FP-1 and TDBC-CPMs. The Rabi splitting of TDBC-FP-1 (1250 cm^{-1}) is in line with most of the TDBC-CPMs and, thus, allow the comparison

of their emission. Moreover, TDBC-FP-1 was excited at an angle of 35°, where the detuning is zero, which is in line again with the conditions of TDBC-CPMs. The emission angle was scanned from 10° to 50° with steps of 5°. The settings for the emission measurements were the same as those used to collect the emission from the CPMs. The excitation wavelengths were scanned from 540 nm to 610 nm with a step of 5 nm, the emission wavelengths were collected until 690 nm with a step of 1 nm, the slits were 2 nm for the excitation and 3 nm for the emission, and the integration time was set to 1 s.

Figure S40a reports the TDBC-FP-1 emission at different exciting wavelengths. The excitation and emission angles were set to 35°, where the detuning is zero. W_{VAS} is indeed maximized when the detuning is zero according to Eq. 2 and 3. The reflectivity curve at 35° is also reported. It presents two minima, at 2 eV and 2.18 eV, corresponding to the LP and UP, respectively, and a maximum at around 2.1 eV, corresponding to the region of the DS. The emission presents a peak at 2.08 eV and a shoulder at 1.97 eV, which can be attributed to the emission from the DS and the LP, respectively. In Figure S40b the emission is rearranged in function of the $E_{exc} – E_{em}$ (cm^{-1}), to be compared with the CPMs emission reported in Figures S16a to S33a. The emission does not show any feature that resembles the Raman spectrum of TDBC (panel below), which is in stark contrast with the CPM emission. Similar results were obtained for all the other emission angles, which range from 10° to 50°. In conclusion, TDBC-FP-1 does not present any VAS signal.

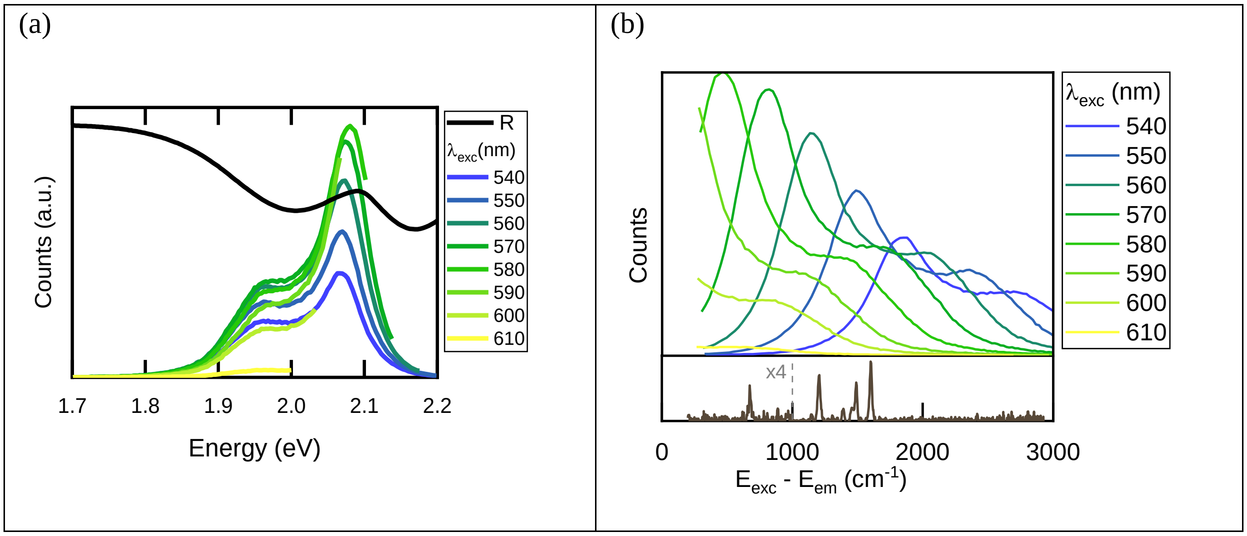

Figure S40: a) Raw emission spectra at different exciting wavelengths of TDBC-FP. The excitation and the emission angles are 35°. The reflectivity of TDBC-FP at 35° is reported as a black line. b) Emission of TDBC-FP as a function of E_{exc}-E_{em}. The Raman spectrum of TDBC is plotted below, with the region below 1000 cm^{-1} scaled by a factor x4. The emission is plotted from λ_{exc} = 540 nm (blue line) to 610 nm (yellow line).

VAS in TDBC-FPs. VAS was not observed in TDBC-FP-1 exciting with the same conditions as TDBC-CPMs, even if VAS was already reported in TDBC-FPs.[1, 2] This contradiction is just apparent because the previous works used a high-intensity laser source rather than a weak Xe lamp as in our study. In order to reproduce the literature results, TDBC-FP-1 was excited with a laser at 532 nm (OBIS, Coherent) and a power of 1 mW. The excitation angle was set to 25° and the emission angle was scanned from 10° to 50° with a step of 5°, resulting in two intense peaks in the region of the DS and the LP (Figure S41a). The sum of the angle-resolved emission was plotted as a function of the energy separation from the DS (Figure S41b, blue area). The emission sum resembles partially the Raman spectrum of TDBC, although this is partially hidden by the DS emission. This is in agreement with the literature, which suggests that VAS signals are much clearer when the TDBC-FPs have higher $\hbar\Omega_R$.[1] For this reason, TDBC-FP-2 was prepared similarly to TDBC-FP-1, but with a higher TDBC concentration (15 mM instead of 4 mM) and a reduced dilution with Millipore water (1.5 instead of 1.7). The molecular layer was then spin-coated at 1300 rpm. The reflectivity of TDBC-FP-2 was fitted with a coupled harmonic oscillator

model (Figure 41c), resulting in $\hbar\Omega_R$ = 370 meV (2984 cm^{-1}). The other results of the fitting were: L_{cav} = 175 nm, n_{eff} = 1.64, and E_{exc} = 2.2 eV. The emission was measured in the same conditions as for TDBC-FP-1 (Figure S41d). The sum of the angle-resolved emission was then plotted as a function of the energy separation from the DS (Figure S41e, blue area). In this case, the emission sum agrees with the Raman spectrum of TDBC. In conclusion, VAS is active in TDBC-FP only with a high-intensity laser source and occurs after a fast relaxation to the DS. On the contrary, VAS in CPMs is active even using low-intensity lamp sources and occurs before any relaxation, because of a reduced number of coupled excitons N.

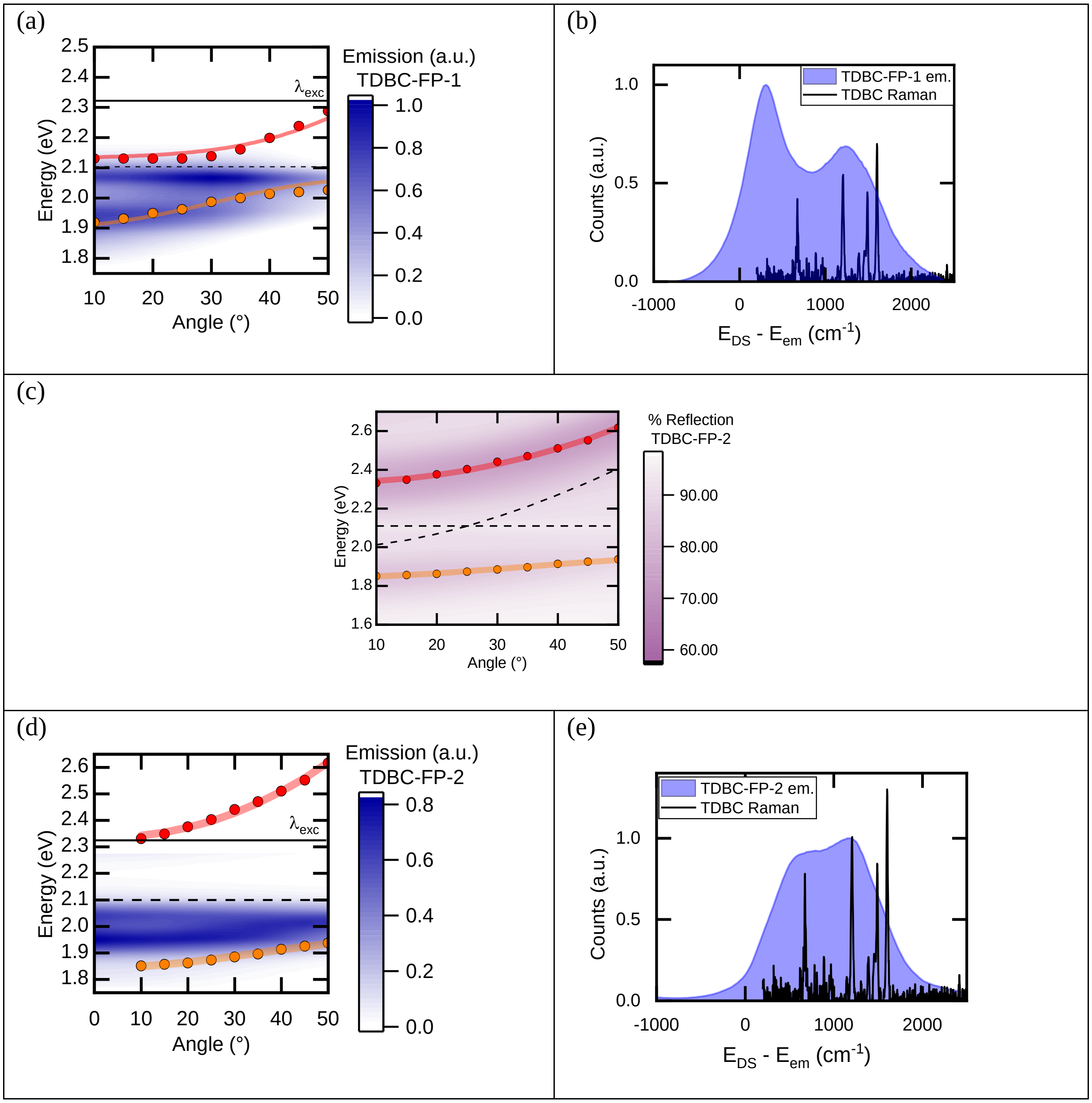

Figure S41: a) Angle-resolved emission of TDBC-FP-1 exciting with a laser source of 532 nm. The minima of the angular resolved reflectivity with the fitted coupled harmonic oscillator model are overlaid over the emission. b) Sum of all the angle-resolved emissions plotted as a function of $E_{DS} – E_{em}$ (blue area) compared with the TDBC Raman spectrum (black line). c) Angle-dependent reflectivity of TDBC-FP-2. The UP and LP maxima are reported as red and orange circles, respectively. The UP and LP branches obtained with the coupled harmonic oscillator model are reported as red and orange lines, respectively. E_{exc} is reported as a horizontal black dashed line, and E_{cav} as a black dashed line. d) and e) are the same as a) and b), respectively, but for TDBC-FP-2.

9. References

(1) Hulkko, E.; Pikker, S.; Tiainen, V.; Tichauer, R. H.; Groenhof, G.; Toppari, J. J. Effect of molecular Stokes shift on polariton dynamics. *J. Chem. Phys.* **2021**, *154* (15), 154303.

(2) Coles, D. M.; Michetti, P.; Clark, C.; Tsoi, W. C.; Adawi, A. M.; Kim, J. S.; Lidzey, D. G. Vibrationally assisted polariton-relaxation processes in strongly coupled organic-semiconductor microcavities. *Adv. Funct. Mater.* **2011**, *21*, 3691-3696.
